# Location of CD39^+^ T cell subpopulations within tumors predict differential outcomes in non-small cell lung cancer

**DOI:** 10.1136/jitc-2023-006770

**Published:** 2023-08-30

**Authors:** Lilian Koppensteiner, Layla Mathieson, Samuel Pattle, David A Dorward, Richard O’Connor, Ahsan R Akram

**Affiliations:** 1 Centre for Inflammation Research, The University of Edinburgh, Edinburgh, UK; 2 Department of Pathology, Royal Infirmary, Edinburgh, UK; 3 Cancer Research UK Edinburgh Centre, Institute of Genetics & Molecular Medicine, The University of Edinburgh, Edinburgh, UK

**Keywords:** tumor biomarkers, biomarkers, tumor, immune checkpoint inhibitors, lung neoplasms, non-small cell lung cancer

## Abstract

**Purpose:**

An improved mechanistic understanding of immunosuppressive pathways in non-small cell lung cancer (NSCLC) is important to develop novel diagnostic and therapeutic approaches. Here, we investigate the prognostic significance of the ectonucleotidases CD39 and CD73 in NSCLC.

**Experimental design:**

The expression and localization of CD39, CD73 and CD103 was digitally quantified in a cohort of 162 early treatment naïve NSCLC patients using multiplex-immunofluorescence and related to patient outcome. Expression among different cell-populations was assessed via flow cytometry. Targeted RNA-Seq was performed on CD4^+^ and CD8^+^ T cells from digested NSCLC tumor tissue and single-cell RNA-Seq data was analyzed to investigate the functional significance of CD39^+^ T cell populations.

**Results:**

We demonstrate that flow cytometry of early untreated NSCLC patients shows an upregulation of CD39 expression in the tumor tissue among natural killer (NK) cells, fibroblasts and T cells. CD73 expression is mainly found among fibroblasts and Epcam+cells in the tumor tissue. Multiplex Immunofluorescence in a cohort of 162 early untreated NSCLC patients demonstrates that CD39 expression is mainly localized in the tumor stroma while CD73 expression is equally distributed between tumor nest and stroma, and high expression of CD39 and CD73 in the tumor stroma is associated with poor recurrence-free survival (RFS) at 5 years. Additionally, we find that CD8+T cells located in the tumor nest express CD103 and the density of CD39+CD103+CD8+ T cells in the tumor nest predicts improved RFS at 5 years. Targeted RNA-Seq shows that the tumor microenvironment of NSCLC upregulates regulatory pathways in CD4^+^ T cells and exhaustion in CD8^+^ T cells, and analysis of a single cell RNA sequencing dataset shows that CD39^+^CD4^+^ cells are enriched in Treg signature gene-sets, and CD39^+^CD103^+^ cytotoxic T lymphocyte show gene signatures indicative of an exhausted cytotoxic phenotype with upregulated expression of CXCL13.

**Conclusions:**

Knowledge of patterns of distribution and location are required to understand the prognostic impact of CD39^+^ T cell populations in NSCLC. This study provides an improved understanding of spatial and functional characteristics of CD39^+^ T cells and their significance to patient outcome.

**WHAT IS ALREADY KNOWN ON THIS TOPIC**

- T-cell subsets play a key role in shaping anticancer responses. A greater understanding of this is critical to develop diagnostic and therapeutic approaches.

**WHAT THIS STUDY ADDS**

- In our work in human lung cancer, we show that CD39^+^ CD4^+^ T cells include a Treg population which predict poor outcome and that CD39^+^CD103^+^CD8^+^ T cells represent a cytotoxic T cell population which confers a survival benefit if high densities are observed in the tumor nest. Moreover, we show that purigenic synthesis in stromal areas confers poor prognosis.

**HOW THIS STUDY MIGHT AFFECT RESEARCH, PRACTICE OR POLICY**

- Our findings highlight the role of CD39^+^ T cell subsets and their spatial distribution in determining prognosis in treatment naïve non-small cell lung cancer undergoing curative resection, bridging the gap between the identification of these phenotypes in vitro and their clinical relevance.

## Introduction

The immune response to cancer depends on efficient cytotoxic T lymphocytes (CTL), conventional CD4^+^ T cells and B cells and checkpoint inhibition therapy is aimed at reinvigorating local cytotoxic T cells. However, the rate of immunotherapy failure in non-small cell lung cancer (NSCLC) is as high as 80% in unselected patients.^1^ Immunogenic hot tumors with high densities of tumor infiltrating lymphocytes (TILs) are linked to improved response to immunotherapy.^2^ Therefore, identifying the extent of cytotoxic lymphocyte infiltration, as a measure of pre-existing immunity could be used to predict clinical outcome or responsiveness to treatment. In recent years, CD39 has emerged as a marker to identify T cells specific for tumor antigen.^3^ Unlike PD1, CD39 expression is lacking in bystander CD8^+^ T cells and highly expressed in CD8^+^ T cells specific for tumor antigens in colorectal cancer and melanoma.^4 5^ CD39 expression on CTL also describes a terminally exhausted population in viral infections and tumors.^4–8^

Duhen *et al* showed that CD39^+^CD8^+^TIL often coexpress the tissue resident memory (TRM) marker CD103.^9^ In head and neck squamous cell cancer (HNSCC) and ovarian cancer, these CD39^+^CD103^+^CD8^+^ cells display an exhausted TRM T cell phenotype that, despite its reduced capacity for effector cytokine production, shows great cytotoxicity against neoplastic cells and can be found across human solid malignancies.^9^ We have previously reported this population in NSCLC and found that CD39 expression is upregulated by crosstalk between cancer-associated fibroblasts (CAFs) and T cells, via TGF-β.^10^ Functionally, CD39 is an outer membrane enzyme that converts extracellular ATP (eATP) into AMP, which is then hydrolysed into adenosine by CD73. Adenosine has highly immunosuppressive effects, as it enhances the activity of suppressive immune cells including tumor associated macrophages, myeloid derives suppressor cells and Tregs, and inhibits neutrophils, NK cells, dendritic cells (DCs) and T cells, which ultimately results in tumor growth and progression.^11^ We aimed to investigate the spatial distribution of cells co-expressing CD39 and CD103 in relation to cells expressing the adenosine generating ectonucleotidase CD73 to better understand where the potential for adenosine-mediated immunosuppression of tumor-specific CTL is greatest. To address this, we characterized CD39, CD73 and CD103 in a cohort of early treatment naïve NSCLC and investigated their functional relevance using a publicly available NSCLC single cell RNA sequencing dataset.^12^ Patients with high CD39 and CD73 expression in close proximity to each other show markedly worse outcome. CD39^+^CD4^+^ T cells which upregulate genes associated with an active Treg phenotype (CD25) also predicts poor outcome while high densities of CD39^+^CD103^+^CD8^+^ T cells in the tumor nest which upregulate cytotoxicity—and tissue residency genes, are linked to improved 5-year survival.

## Results

### The tumor microenvironment of NSCLC exhibits upregulated expression of CD39

**Figure 1:**
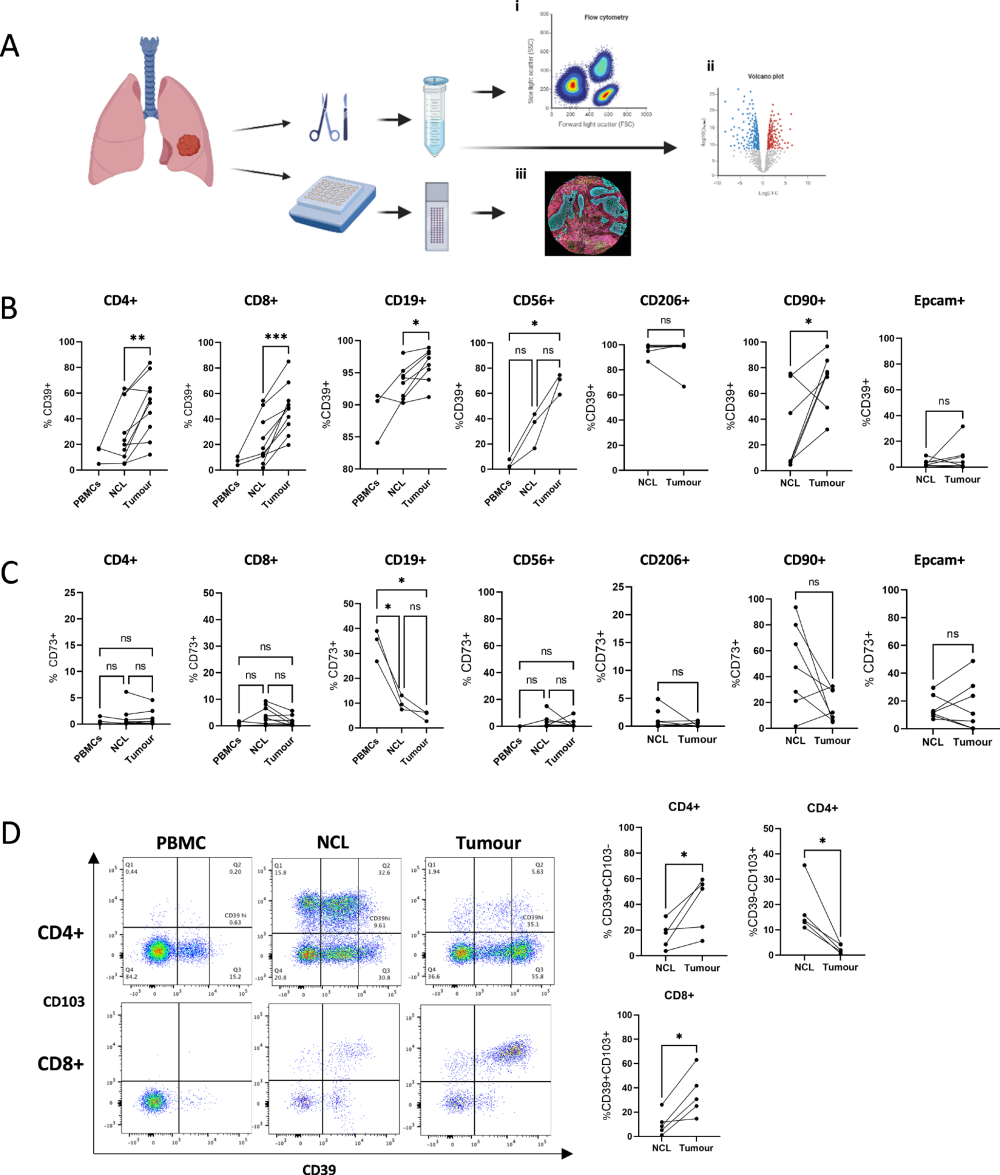
The TME of NSCLC exhibits upregulated expression of CD39. (A) Study design. (B) Expression levels of CD39 shown as % positive within subpopulations of CD4^+^, CD8^+^, CD19^+^ and CD56^+^ cells from peripheral blood, NCL and tumor tissue and CD206^+^, CD90^+^ and Epcam^+^ cells from NCL and tumor tissue of early stage NSCLC patients. (C) Expression levels of CD73 shown as % positive within subpopulations of CD4^+^, CD8^+^, CD19^+^ and CD56^+^ cells from peripheral blood, NCL and tumor tissue and CD206^+^, CD90^+^ and Epcam^+^ cells from NCL and tumor tissue of early NSCLC patients. (D) Representative staining of CD4^+^ and CD8^+^ T cells from a paired PBMC, NCL and tumor sample. Frequency of CD39^+^CD103^−^ and CD39^−^CD103^+^ CD4^+^ T cells and CD39^+^CD103^+^ CD8^+^ T cells. Paired t-tests were used for statistical analysis of two groups, one-way ANOVAs with Tukeys multiple comparisons were used for comparing 3 groups. (*p<0.05, **p<0.01, ***p<0.001). Images created with Biorender.com. ANOVAs, analysis of variances; NCL, non-cancerous lung; NSCLC, non-small cell lung cancer; PBMC, peripheral blood mononuclear cell; TME, tumor microenvironment.

All patients we report on are treatment naïve and underwent surgical resection with an aim for curative intent. We first analyzed the expression of CD39, CD73 and CD103 in peripheral blood and non-cancerous lung (NCL) and tumor tissue of early untreated NSCLC using flow cytometry (figure 1A). Peripheral lymphocyte CD39 expression in NSCLC patients was limited to B cells (CD19^+^), while low levels of CD39 expression were observed among CD4^+^ T cells, CD8^+^ T cells and NK cells (CD56^+^) (figure 1B). No differences were observed in comparison to CD39 expression in the peripheral blood of healthy volunteers (online supplemental figure S1). In NSCLC patients, we find an increase of CD39^+^ cells in NCL tissue compared with levels in peripheral blood, which was further elevated in the tumor microenvironment (TME) of NSCLC among CD4^+^ and CD8^+^ T cells, in line with published reports of multiple human solid tumors.^8 13–17^ We additionally observed an upregulation of CD39 expression on NK cells and fibroblasts, identified as CD90^+^ cells in tumor compared with NCL tissue. Macrophages (CD206^+^ cells) are equally CD39^+^ in NCL and tumor tissue (figure 1B).

10.1136/jitc-2023-006770.supp1 Supplementary data

Considering the downstream processing of CD39 synthesized cAMP into adenosine by CD73, we also analyzed CD73 expression in NSCLC to further investigate which cell types contribute to adenosine synthesis in the TME of NSCLC. We observed high frequencies of CD73 expression among CD19^+^ cells and CD8^+^ T cells in the peripheral blood of healthy donors which was significantly reduced in the peripheral blood of early NSCLC patients (online supplemental figure S2). This could reflect a lower proportion of naïve CD8 T cells in those patients as naïve cells are the main cells expressing CD73 in the CD8 compartment. Notably, some patients exhibited high levels of CD73 expression in CD90^+^ cells of NCL tissue, which was reduced in cancerous tissue. EPCAM^+^ cells display similar levels of CD73 expression in both NCL and tumor tissue (figure 1C). Representative flow cytometry staining for CD39 and CD73 is shown in online supplemental figure S3.

While CD39 expression is often associated with Tregs, their coexpression of CD73 is controversial.^18^ Gourdin *et al* have shown that in human breast cancer, Tregs are CD39^+^ and CD73−, and instead found CD73 expression in a subset of effector CD4^+^ T cells.^19^ Similarly, we confirm that FOXP3^+^ are CD39^+^ (online supplemental figure S4A)^10 13^ and we see a significant enrichment of CD39^+^Foxp3^+^ cells in the tumor tissue (online supplemental figure S4B), yet see little CD73 expression among CD4^+^ T cells in the tumor (figure 1C).

Our data illustrate that multiple different cell types upregulate CD39 expression in the cancerous tissue of NSCLC and are therefore able to contribute to the production of adenosine. In stark contrast, we did not observe an upregulation of CD73 in the TME within cell types tested and saw highest frequencies among fibroblasts and EPCAM^+^ cells, suggesting that adenosine production is likely induced by non-immune cells.

As previously shown,^9 10^ the majority of CD39^+^ CD8^+^ TIL coexpress the TRM marker CD103. Conversely, among CD4^+^ T cells, we do observe a CD103^+^CD39^−^, as well as a CD103^+^CD39^+^ population in NCL tissue, however, these are depleted in the cancerous tissue, where we see a shift toward a single positive (SP) CD39high population (figure 1D). CD39^+^CD4^+^ T cells include FOXP3^+^ Tregs and FOXP3^−^ conventional T cells (online supplemental figure S4C).

CD19^+^ cells, CD56^+^ cells and CD206^+^ cells display low frequencies of CD103, suggesting CD103 is exclusive to T cells in the lung tissue (online supplemental figure S5).

### CD39, CD103 and CD73 display distinct spatial signatures in NSCLC

**Figure 2:**
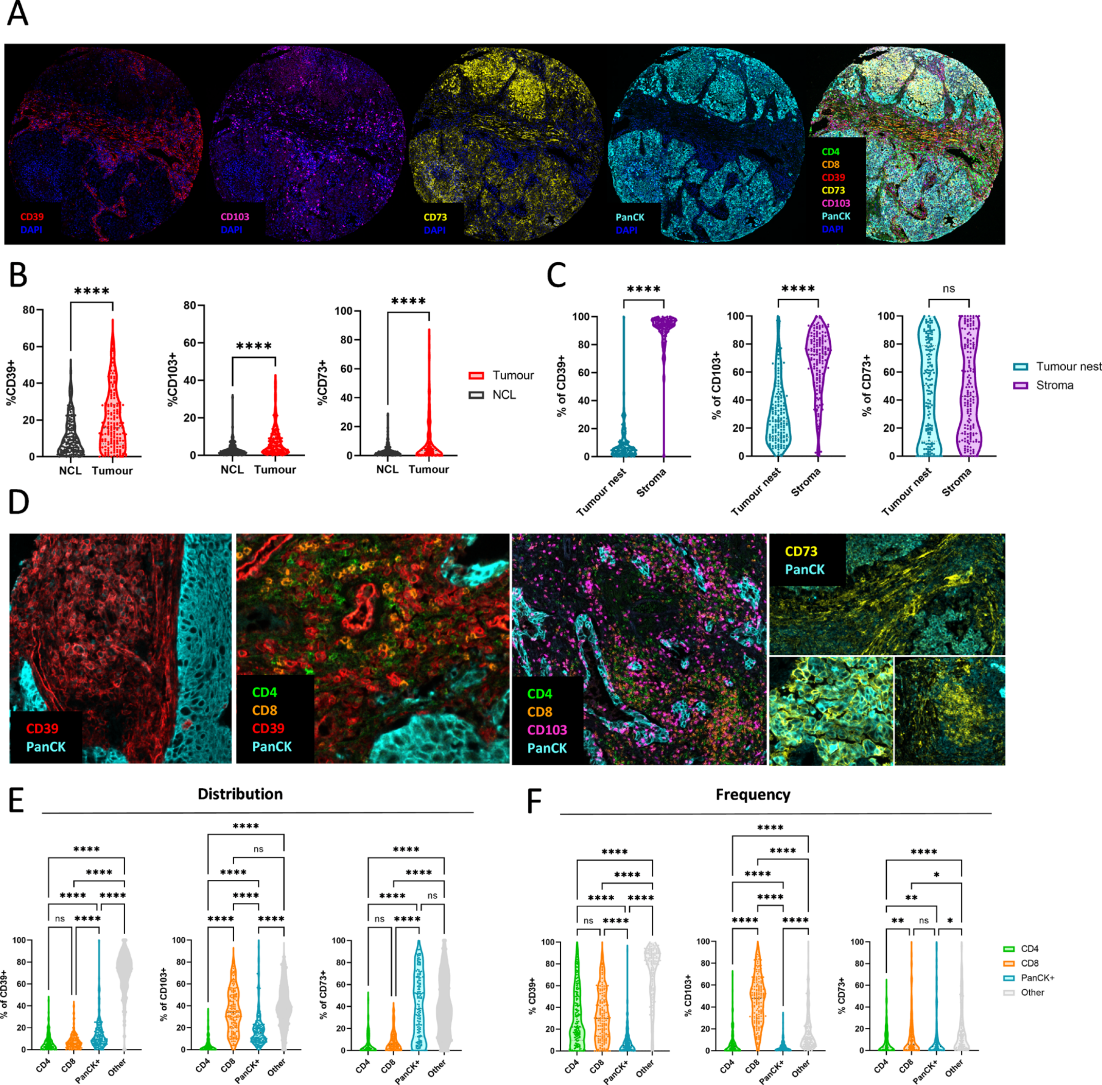
CD39, CD103 and CD73 display distinct spatial signatures in NSCLC. (A) Representative staining patterns of CD39, CD103, CD73 and PanCK in a tumor tissue sample. (B) Frequency of CD39, CD103 and CD73^+^ cells in NCL and tumor tissue samples. N=162 (C) Distribution of CD39^+^, CD103^+^ and CD73^+^ cells across tumor nest and stroma. N=162 (D) Representative cell staining patterns of CD39, CD103 and CD73 in tumor tissue samples. (E) Distribution of CD39^+^, CD103^+^ and CD73^+^ cells across celltypes PanCK^+^, CD4^+^ and CD8^+^ cells. (F) Frequency of CD39^+^, CD103^+^ and CD73^+^ cells among PanCK^+^, CD4^+^ and CD8^+^ cells. Unpaired t-tests were used to compare NCL and Tumor groups. N=162. Paired t-tests were used to compare tumor nest and stroma groups. Two-way ANOVAs were used to compare distribution across celltypes. (*p<0.05, **p<0.01, ***p<0.001). ANOVAs, analysis of variances; NCL, non-cancerous lung; NSCLC, non-small cell lung cancer.

To assess the localization of surface markers involved in adenosine production as well as T cell subpopulations characterized by CD39 and CD103 expression, we investigated their presence and spatial organization in FFPE tissue of 162 early treatment naive NSCLC patients with multiple histological subtypes (online supplemental table S1). CD39 expression was locally elevated in the tumor tissue compared with NCL (figure 2A,B, online supplemental figure S6) where the vast majority was expressed in the tumor stroma (figure 2C,D). The distribution of CD39^+^ cells across cell types shows that only a small fraction of CD39^+^ cells are CD4^+^ or CD8^+^ T cells (figure 2E) and we find high frequencies of CD39^+^ cells within CD4^+^ T cells, CD8^+^ T cells as well as other (non-CD4 non-CD8 non-PanCK) cells (figure 2F), confirming the presence of other CD39^+^ cells as shown by flow cytometry (figure 1B). Conversely, CD103 was expressed in both tumor and stroma areas (figure 2C) and mainly expressed by CD8^+^ T cells (figure 2E), with highest frequencies in CD8^+^ T cells (figure 2F). CD73 expression was found in both tumor area staining up to 100% of cancer cells (figure 2A) and in the tumor stroma (figure 2C) staining CD4^+^ and CD8^+^ T cell, as well as other (non-CD4 non-CD8) cells (figure 2D,E), which is in line with our flow cytometry data showing that the source of CD73 can come from multiple different cell types including immune cells, EPCAM^+^ cells and fibroblasts (figure 1C). The observed staining patterns of CD39 and CD73 in the multiplex immunofluorescence (mxIF) suggest that close proximity of CD39 and CD73 likely occurs in the tumor stroma.

### CD103^−^CD8^+^ T cells are unable to infiltrate the tumor nest

**Figure 3:**
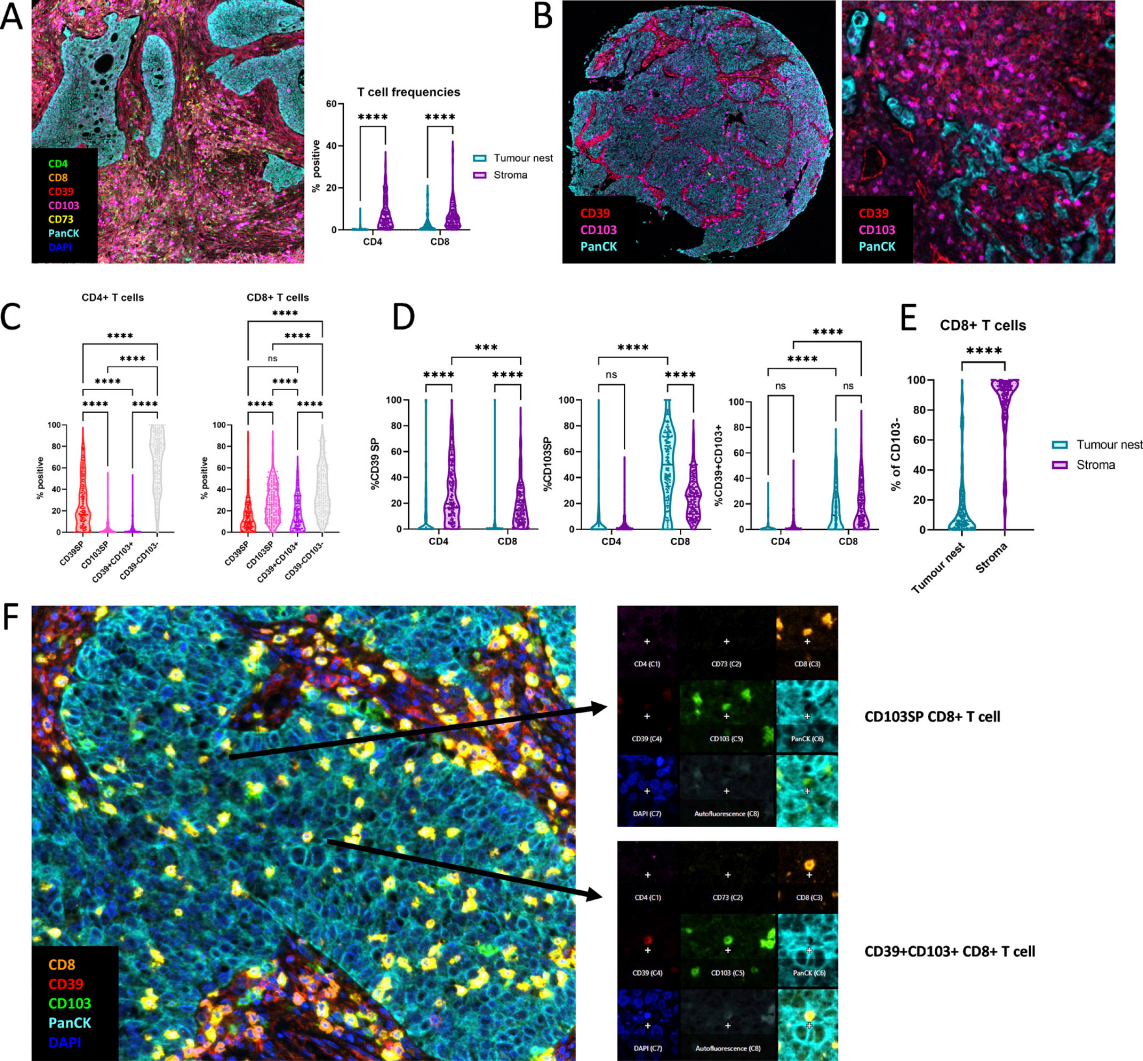
Infiltration of T cell subsets into the tumor nest of NSCLC. (A) Representative image of T cell restriction to the stroma and frequency of CD4^+^ and CD8^+^ T cells in tumor nest and stroma. N=162 (B) Representative images showing CD103 and CD39 expression. (C) Frequency of CD39SP, CD103SP, CD39^+^CD103^+^ and CD39^-^CD103^-^ cells within CD4^+^ and CD8^+^ T cells. (D) Frequency of CD39SP, CD103SP and CD39^+^CD103^+^ within CD4^+^ and CD8^+^ T cells in tumor nest and stroma area. N=162 (E) Distribution of CD103 negative CD8^+^ T cells across tumor nest and stroma. N=162 (F) Example image showing a CD103SP CD8^+^ TIL (top) and a CD39^+^CD103^+^ CD8^+^ TIL (bottom) in all eight isolated fluorescent channels. Two-way ANOVAs were used to compare groups. ***p<0.001. ANOVA, analysis of variances; NSCLC, non-small cell lung cancer; TIL, tumor infiltrating lymphocyte.

At cancerous tissue sites, the level of T cell infiltration was markedly elevated compared with NCL tissue (online supplemental figure S7A), however, the majority of CD4^+^ and CD8^+^ T cells were confined to stromal areas (figure 3A). CD4^+^ T cells are mainly CD39 SP and CD39^−^CD103^−^. Among CD8^+^ T cells, we observe populations of CD39SP and CD39^+^CD103^+^ cells, and high frequencies of CD103SP and CD39^−^CD103^−^ cells (figure 3C). Overall, the distribution of all subsets of CD4^+^ and CD8^+^ T cells shows that the majority of cells are found in the tumor stroma (online supplemental figure S8). While a higher frequency of CD39SP CD8^+^ T cells was found in the stroma, the frequency of CD39^+^CD103^+^ cells among CD8^+^ T cells was equal in tumor nest and stroma and notably, the highest frequencies of CD103SP cells among CD8^+^ T cells were observed inside of the tumor nest (figure 3B,D) Immunofluorescence staining patterns illustrate that CD8^+^ T cells in the tumor nest are typically CD103^+^ and indeed, CD8^+^ T cells that did not express CD103 were restricted to the stromal area (figure 3E). This suggests that bystander CD8 TRM are recruited to the tumor nest due to their CD103 expression or that tumor-reactive CD8 T cells upregulate CD103 in the tumor following TCR stimulation in a TGF-b-rich environment.^20^

### CD39^+^ T cells upregulate pathways of T cell exhaustion

**Figure 4:**
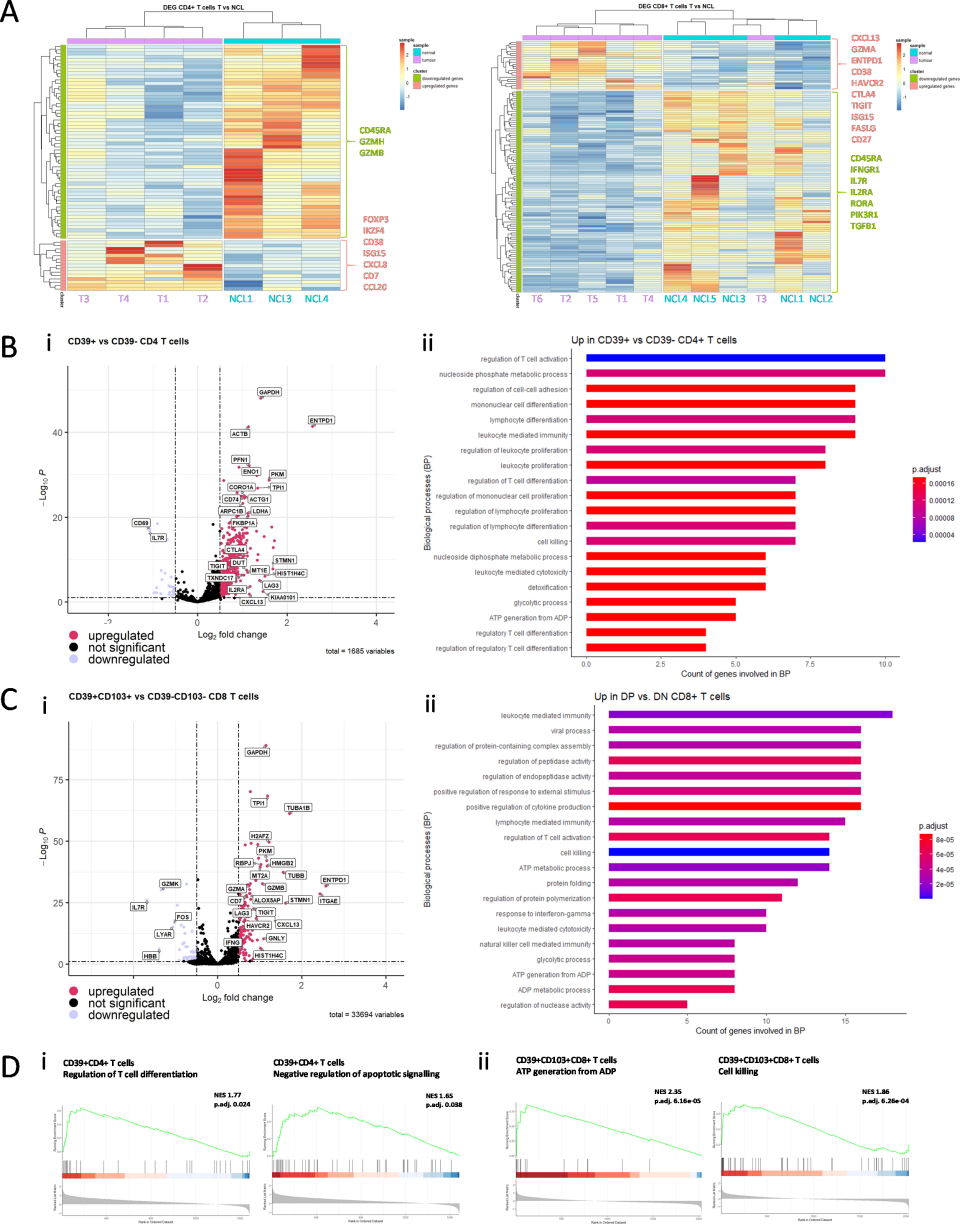
Transcriptomic and functional analysis of T cell subsets in NSCLC. (A) Heat maps showing unsupervised clustering of DEGs from Nanostring gene expression data of CD4^+^ (left) and CD8^+^ (right) T cells isolated from digested NCL and tumor tissue. (B) (i) Volcano plot showing DEG between CD39^+^ and CD39- CD4^+^ T cells. (ii) Bar plots of enrichment p values and gene count of respective gene sets of top 20 GO biological processes upregulated in CD39^+^ CD4^+^ T cells compared with CD39-CD4^+^ T cells. (C) (i) Volcano plot showing DEG between CD39-CD103- and CD39^+^CD103^+^ CD8^+^ T cells. (ii) Bar plots of enrichment p values and gene count of respective gene sets of top 20 GO biological processes upregulated in CD39-CD103^+^ CD8^+^ T cells compared with CD39-CD103- CD8^+^ T cells. (D) (i) GSEA of DEG in CD39^+^ CD4^+^ T cells compared with CD39-CD4^+^ T cells in regulation of T cell differentiation gene set (GO:0045580) and negative regulation of apoptotic signaling gene set (GO:2001234). (ii) GSEA of DEG in CD39^+^CD103^+^CD8^+^ T cells compared with CD39-CD103-CD8^+^ T cells in ATP generation from ADP (GO:0006757) and cell killing (GO:0001906) gene sets. GO, gene ontology; GSEA, gene set enrichment analysis; NCL, non-cancerous lung; NSCLC, non-small cell lung cancer.

We next performed Nanostring nCounter gene expression analysis of sorted CD4^+^ and CD8^+^ T cells from NCL and tumor tissue from early untreated NSCLC patients to analyze the effect of the TME on the T cell transcriptome. CD4^+^ T cells isolated from tumor tissue downregulate granzymes B and H and upregulate genes associated with regulatory T cells (FOXP3, IKZF4, CD38, ISG15). Notably, CD8^+^ T cells downregulate CD45RA and IL7R (suggesting loss of their naïve state) and metabolism genes (RORA, PIK3R1) indicating the suppressive effect of the TME on CD8^+^ T cell function. Furthermore, the CD39 encoding gene ENTPD1 is among the most highly upregulated genes in CD8^+^ T cells isolated from tumor tissue, along with other T cell exhaustion marker encoding genes (HAVCR2, CTLA4, TIGIT) (figure 4A, online supplemental figures S9 and 10).

To further investigate the functional role of CD39^+^CD4^+^ and CD39^+^CD103^+^ CD8^+^ T cell subsets, we used a publicly available dataset^12^ of single-cell RNA sequencing of NSCLC tumor tissue. CD39^+^CD4^+^ T cells downregulate CD69, in line with reduced activation, and upregulate genes associated with exhaustion (CTLA4, LAG3, TIGIT) (figure 4Bi). Both Gene ontology (GO) and gene set enrichment analysis (GSEA) show a significant enrichment in gene sets of regulation of T cell activation, differentiation, proliferation and negative regulation of apoptotic signaling pathway genes further illustrating that CD39^+^CD4^+^ T cells include a particularly suppressive regulatory T cell population (figure 4Bii,Di).

In comparison to CD39^−^CD103^−^ CD8^+^ T cells, CD39^+^CD103^+^ CD8^+^ T cells downregulate IL7R and the early activation marker CD69, and upregulate exhaustion markers (LAG3, TIGIT, HAVCR2), however, we also see an upregulated expression of IFNG and granzyme A and B indicative of effector function and high cytotoxicity (figure 4Ci). GO and GSEA analysis indicate a link to leukocyte mediated cytotoxicity, cell killing, and genes associated with ATP generation (figure 4Cii,Dii). More specifically, in comparison to CD103SP CD8^+^ T cells, CD39^+^CD103^+^ CD8^+^ T cells upregulate genes associated with positive regulation of cytokine production and type I interferon production and cell killing (online supplemental figure S11 file 1), suggesting that CD39 expression likely enriches for tumor-reactive cytotoxic T cells.

Interestingly, both CD39^+^ populations (figure 4B,C) show upregulated expression of the tissue resident marker ALOX5AP as well as CXCL13 expression, which has been described to be a feature of exhausted TILS and CD103^+^ TILS in cancer,^13 21^ co-occurring with B cell recruitment and beneficial tertiary lymphoid structure (TLS) formation, in line with our flow data showing increased B cell frequencies in the TME of NSCLC (online supplemental figure S12).

### Spatial patterns of distinct T cell populations predict outcome in NSCLC

**Figure 5:**
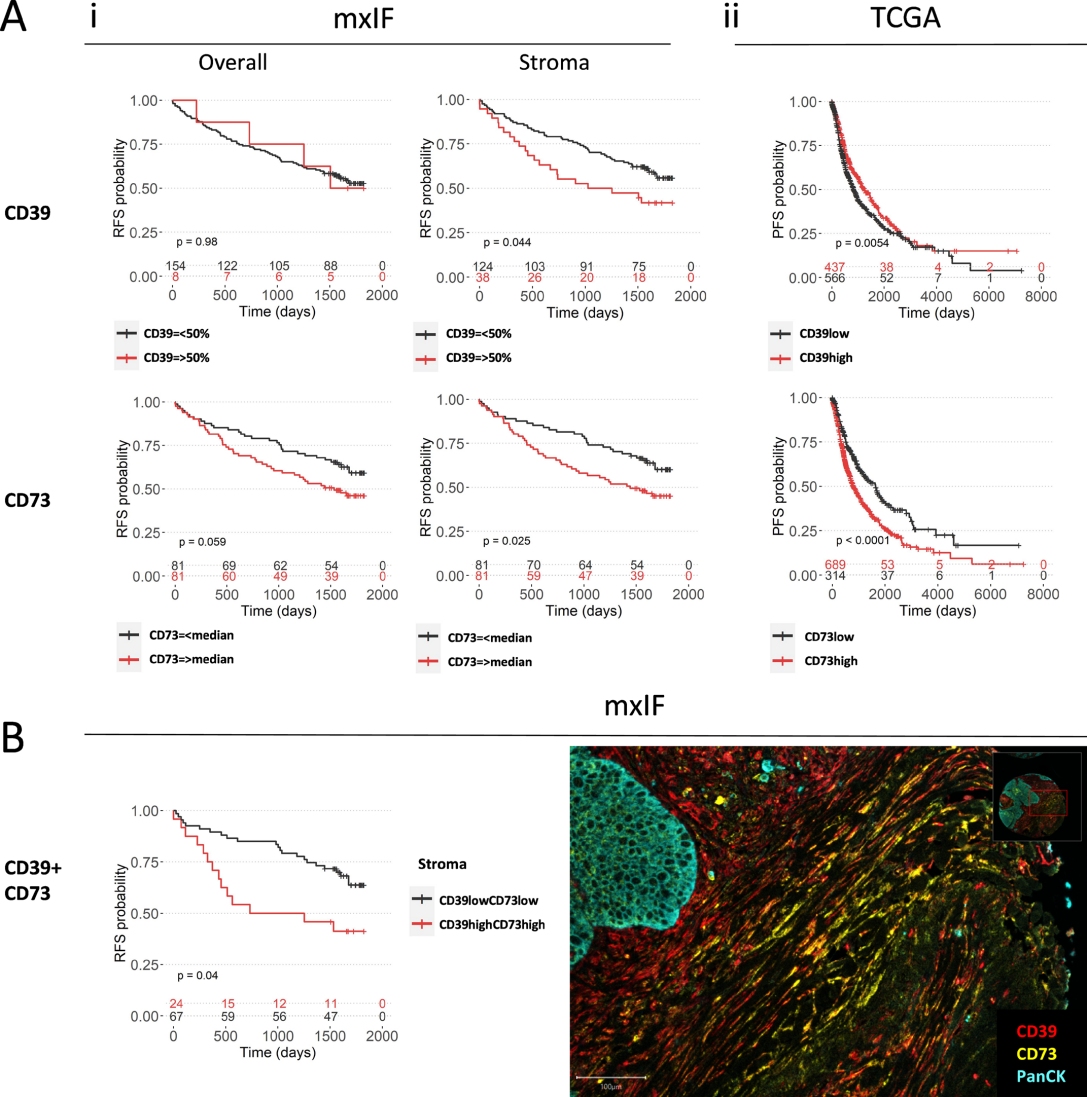
Spatial patterns of CD39 and CD73 predict outcome in NSCLC (A) Kaplan-Meier curves showing (i) 5-year RFS based on stromal and intratumoral expression of CD39 (top panel) and CD73 (bottom panel) of mxIF data of early untreated NSCLC patients (n=162) and (ii) 19 year PFS probability based on high/low CD39 (top) and CD73 (bottom) of TCGA data. (B) 5-year RFS based on combined stromal CD39 and CD73 expression of mxIF data (left) and representative image of stromal CD39 and CD73 staining in tumor tissue of NSCLC (right). Log Rank tests were used for survival analysis. mxIF, multiplex immunofluorescence; NSCLC, non-small cell lung cancer; PFS, progression-free survival; RFS, recurrence-free survival; TCGA, The Cancer Genome Atlas.

We hypothesized that CD39 and CD73, as enzymes responsible for adenosine synthesis, could negatively affect patient outcome. While overall CD39 expression in our mxIF dataset does not affect recurrence-free survival (RFS), frequency of CD39^+^ cells present in the stroma predicts reduced RFS probability at 5 years (figure 5Ai). Overall expression of CD73 has a negative predictive effect which is more pronounced when CD73 is expressed in the stroma (figure 5Ai). This observation is independent of PanCK^+^ frequency (online supplemental table S2C). Moreover, a combination of high stromal CD39 and CD73 predicts reduced RFS probability (figure 5B). While non-significant, there is a similar trend for overall survival (OS) (online supplemental figure S13). Using TCGA data, we similarly see that, independent of age, gender, smoking and pathological stage (online supplemental table S3), CD73 predicts reduced long-term progression-free survival (PFS) probability (figure 5Aii), and OS (online supplemental figure S13) as previously shown by Gao *et al*.^22^ Interestingly, overall CD39 expression in the TCGA data confers improved long-term PFS (figure 5Aii) and OS (online supplemental figure S13) perhaps due to its high expression levels among immune cells and tumor specific CD8^+^ T cells.

**Figure 6:**
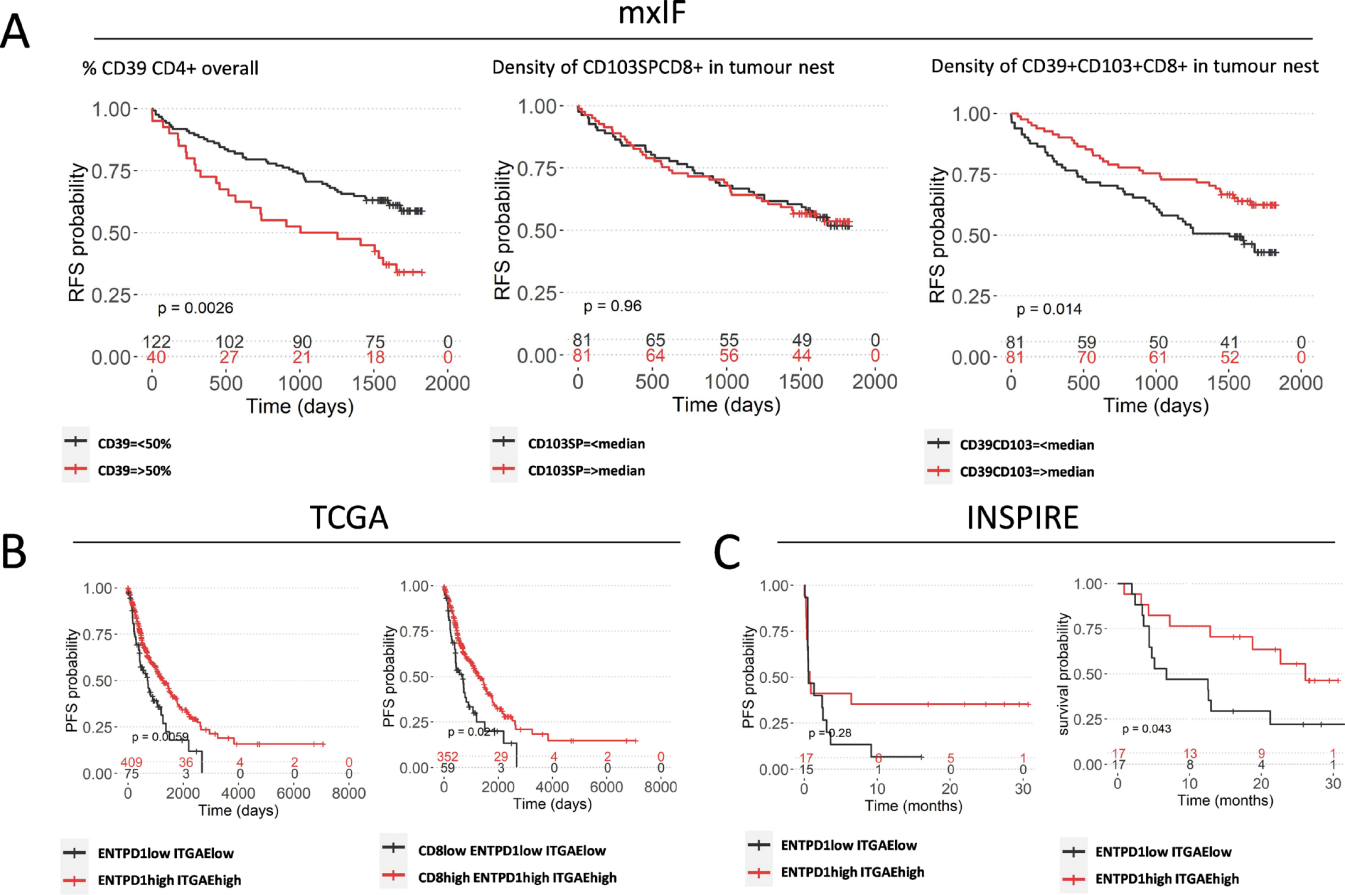
CD39^+^ T cell populations predict outcome in NSCLC Kaplan-Meier curves showing (A) 5-year RFS based on frequency of CD39^+^ within CD4^+^ T cells overall (left) and density of CD103SP CD8^+^ T cells (center) and CD39^+^CD103^+^ CD8^+^ T cells in the tumor nest (right). (B) 19-year PFS probability based on high/low ENTPD1 and ITGAE (left) and CD8, ENTPD1 and ITGAE (right) of TCGA data. (C) PFS (left) and OS (right) following three cycles of anti-PD1 therapy of the INSPIRE trial based on gene expression of ENTPD1 and ITGAE at baseline prior to therapy. Log rank tests were used for survival analysis. mxIF, multiplex immunofluorescence; NSCLC, non-small cell lung cancer; OS, overall survival; PFS, progression-free survival; RFS, recurrence-free survival; TCGA, The Cancer Genome Atlas.

To assess this, we investigated predictive value of distinct T cell subpopulations considering their spatial signatures as identified by our mxIF dataset. We see a marked reduction of RFS probability in patients with high CD39 expression among CD4^+^ T cells overall, in line with their regulatory features and phenotypic traits of highly suppressive Tregs (figure 6A), which was found to be independent of overall CD4 frequency. (online supplemental table S4A) In stark contrast, the density of CD39^+^CD103^+^ CD8^+^ T cells in the tumor nest significantly predicts favorable RFS probability, independent of CD8^+^ T cell density (online supplemental table S4B), while their presence overall or in stromal areas does not (online supplemental figure S14). Moreover, the density of bystander CD103SP CD8^+^ T cells does not predict improved outcome (figure 6A). Notably, the frequency of CD39 SP CD8^+^ T cells in the tumor nest is too low to conduct robust analysis. These observations were broadly independent of tumor size, sex, age, adjuvant chemotherapy and smoking (online supplemental tables S2 and S4) and similar observations were made for OS (online supplemental figure S15A). Furthermore, analysis of TCGA data shows improved long-term PFS probability (figure 6B) and OS probability (online supplemental figure S15B) in patients with high ENTPD1 and ITGAE expression. This illustrates prediction of outcome dependent on CD39 location and cellular subtype. It further demonstrated that functional characteristics of CD39^+^CD103^+^ CD8^+^ T cells (which harbor high cytotoxicity and potential to kill autologous cancer cells in vitro) have a significant biological relevance in the tumor nest in situ which ultimately affects patient outcome in a cohort of early untreated NSCLC patients. Finally, we aimed to investigate whether this population is predictive of response to ICB using the INSPIRE trial (Investigator-initiated Phase II Study of Pembrolizumab Immunological Response Evaluation), a phase II clinical trial of the anti-PD1 pembrolizumab in multiple advanced solid tumors. Although gene expression levels of a single marker (ENDPD1 or ITGAE alone) did not significantly differ across subgroups of pembrolizumab response or pre-therapy and post-therapy (online supplemental figure S16), we found that patients with above median ENTPD1 and ITGAE expression prior to treatment have an increased OS probability following three cycles of pembrolizumab, and a similar, non-significant trend was observed regarding PFS (figure 6C).

## Discussion

Investigating pathways of immune suppression is essential to develop new targets for immunotherapy. In this study, we report that surface enzymes involved in the adenosine pathway are predictive of poor outcome in early NSCLC. CD39 is expressed by multiple immune and non-immune cell types in the TME of NSCLC and mainly localized in the stroma. CD39 expression among T cells can signify several populations, including CD39^+^CD103^−^CD4^+^ T cells associated with transcriptomic signatures of a highly suppressive regulatory phenotype predictive of poor outcome and exhausted yet highly cytotoxic CD39^+^CD103^+^ CD8^+^ T cells which are able to infiltrate the tumor nest where their presence is linked to improved overall and RFS at 5 years in a cohort of early treatment naïve NSCLC patients.

T cell exhaustion is a key feature of CTL within the TME. The exhaustion marker CD39 is highly expressed in terminally exhausted CD8^+^TIL across solid tumors, including NSCLC, where it allows the distinction of tumor-reactive CTL from bystander CD8^+^TIL.^4^ Duhen *et al* discovered that coexpression of CD39 and CD103 identifies exhausted TRM cells based on gene transcripts typical of T cell exhaustion a gene signature associated with TRM and reduced expression of T cell recirculation genes shown by transcriptomic analysis of these populations in HNSCC and ovarian cancer.^9^ In lung cancer, Chow *et al* have shown that CD39 expression identifies tumor-reactive CD8+TILs and demonstrate an association between a high baseline level of CD39+CD8+ T cells and response to ICB monotherapy in a cohort of 23 stage IV lung cancer patients.^3^ Here, we report that the TME of NSCLC highly upregulates the CD39 encoding gene ENTPD1 in CD8^+^ T cells. Flow cytometry analysis shows a shift toward CD39^+^CD103^+^ coexpressing CD8^+^ T cells in tumor tissue compared with NCL, and transcriptomic analysis of single cell RNA Sequencing data demonstrates that these CD39^+^CD103^+^ CD8^+^ T cells are enriched in other exhaustion markers yet display a gene signature indicative of high cytotoxicity and cell killing.^23^ In vitro, CD39^+^CD103^+^CD8^+^ T cells are able to kill autologous tumor cells, while negligible tumor cell killing is observed in CD39^−^CD103^+^ and CD39^−^CD103^-^T cells.^9^ In lung cancer, CD103^+^CD8^+^ T cells increase on immunotherapy and are enriched in responders to anti-PD1.^24^ Similarly, a recent study in breast cancer demonstrates that CD39^+^CD103^+^ T cells are tumor specific, predict improved patient outcome in triple negative breast cancer and demonstrate that this cell population is responsive to immune checkpoint inhibition in vitro.^25^

We have extended these findings to show that in NSCLC, CD8^+^ T cells are confined to the stroma unless they express CD103 in which case they are able to infiltrate the tumor nest. Notably, the density of CD39^+^CD103^+^ CD8^+^ T cells in the tumor nest is linked to improved survival and RFS, while the density of CD103SP CD8^+^ T cells is not, further implying that CD39^+^CD103^+^ CD8^+^ T cells are the main tumor cell killing population in situ. Interestingly, CD39^+^CD103^+^ CD8^+^ T cells are particularly found in malignancies that are immunogenic and responsive in immunotherapy such as triple-negative breast cancer, melanoma and colon cancer, further suggesting that as they are neoantigen specific and cytotoxic, they are primed to respond to PD1 blockade and could offer a target to stratify patients for checkpoint inhibition.^9 26^ In support of this hypothesis, our pan cancer analysis of a phase II clinical trial of pembrolizumab from Cindy Yang *et al* 27 illustrates that high ENTPD1 and ITGAE expression levels prior to treatment predict improved survival probability following anti-PD1 therapy.

In this study, we also report high levels of CD39 among tumor infiltrating CD4^+^ T cells via flow cytometry. This is reflective of the divergent pattern of CD103 expression in CD4^+^ and CD8^+^ populations. Notably CD103^+^CD4^+^TRM are highly reduced in the tumor tissue compared with NCL. Within the tumor infiltrating CD4^+^ population CD39 expression is found on both conventional T cells and Foxp3^+^ Tregs. CD39^+^ Tregs represent a potently suppressive Treg population with increased stability under inflammatory conditions.^16 28–31^ The majority of CD39^+^ CD4^+^ T cells in the TME of NSCLC are confined to the tumor stroma and a high frequency of CD39^+^CD4^+^ T cells predicts poor outcome. Functionally, we report an upregulation of LAG3, CTLA4 and TIGIT in line with the exhausted phenotype and gene signatures of regulatory activity of T cell activation, proliferation, and differentiation.

Interestingly, we observed an increased expression of CXCL13 in both CD39^+^CD4^+^ T cells and CD39^+^CD103^+^ CD8^+^ T cells. Recently, single-cell analysis has identified CXCL13^+^ CD8^+^ T cells as a tumor reactive population correlated to favorable response to ICB.^32^ CXCL13 is a chemoattractant which recruits CXCR5^+^ B cells. In agreement with this we observe a dramatic increase in B cell frequencies in the TME of NSCLC versus B cell recruitment has been associated with TLS formation and a beneficial effect for tumor immunity and responsiveness to checkpoint therapy.^33–36^

Overall, we show that the TME of NSCLC upregulates CD39 expression in multiple cell types including NK cells (CD56^+^), B cells (CD19^+^) and fibroblasts (CD90^+^ cells) suggesting a shared trigger such as TGF-β and hypoxia potentiating CD39 expression.^11^ While CD39 is mainly expressed in stromal areas, CD73 is found in both tumor nest and stroma. This suggests that coordinated activity of CD39 and CD73 is likely to occur in the stroma, resulting in high levels of adenosine in this area, providing a barrier to suppress immune cells prior to their entry into the tumor nest. In support of this, high stromal CD39 and CD73 expression predicts poor RFS at 5 years compared with tumors with low stromal CD39 and CD73 expression.

There is increasing interest in the role of stromal cells in tumor immunity.^37–40^ Our data illustrate CD39 and CD73 expression on stromal cells with characteristic shape of fibroblasts and which are in close proximity to T cells. We have previously described bidirectional upregulation of CD39 and CD73 elicited by CAF—T cell crosstalk.^10^ CAFs can display an autocrine feedforward loop of adenosine production via CD73 and A2B receptors,^41^ and synthesize more adenosine in the presence of activated CD39^+^ T cells.^42^ Thus, this crosstalk could be a potent source of adenosine in the TME.

In conclusion, our data suggest that tumor reactive CD39^+^CD103^+^ CD8^+^ T cells can infiltrate the tumor nest where they confer a survival benefit. However, most CD39^+^ CD8^+^ T cells are trapped in the tumor stroma where we see highest frequencies of CD39^+^CD4^+^ T cells and CD73^+^ stromal cells and therefore likely undergo adenosine mediated immunosuppression. Considering their significant role in the adenosine axis and multiple other immunosuppressive mechanistic roles in the TME, targeting CAFs could simultaneously reduce the adenosine mediated immune suppression and allow redistribution of T cells into the tumor nest (as shown in a preclinical study),^43^ perhaps leading to an increased frequency of CD39^+^CD8^+^ T cells in the tumor nest which we show here improves survival.

## Methods

### NSCLC tissue digest

NSCLC was identified by the pathologist with paired tumor and NCL tissue taken from the same lobe of lung in fresh curative cancer resections. As previously described in O’Connor *et al*,^10^ samples were mechanically minced, spun at 350 g for 5 min, supernatant removed and samples digested with 1 mg/mL Collagenase IV (Merck) and 1 mg/mL DNAse (Merck) in Media for 1 hour at 37°C (with vortexing every 10 min). Digested samples were passed through 70 µm filters, followed by centrifugation at 350 g for 5 min. Supernatants were removed and red blood cells were lysed with red blood cell (RBC) lysis buffer (Biolegend). Cells were washed again, counted, and stained for flow cytometry.

### Peripheral blood mononuclear cell extraction

Blood samples from early-stage NSCLC patients were paired to respective NCL and tumor tissue samples. Healthy donor blood samples were donated by healthy volunteers at the Centre for inflammation Research in the Queen’s Medical Research Institute in Edinburgh. Peripheral blood mononuclear cells were isolated from whole blood using lymphoprep (Stemcell technologies) and SepMate tubes for density gradient centrifugation (Stemcell technologies). Cells were washed, centrifuged at 350 g for 5 min, and stained for flow cytometry.

### Flow cytometry

Cells were washed with 2 mL of serum free DPBS and spun at 350 g for 5 min. The supernatant was removed, and this step was repeated. Cells were stained with Zombie Live Dead UV (Biolegend) (1 µL per test in PBS) to label dead cells and incubated for 30 min at room temperature in the dark. The reaction was stopped by adding two mls FACS buffer (DPBS+2% FCS) to each tube, followed by centrifugation at 350 g for 5 min and removal of the supernatant. Prior to surface marker staining, cells were treated with FC block for 5–10 min at room temperature, to reduce non-specific binding (5 µL/test in 50 µL FACS buffer). Antibodies were prepared at the required concentration in FACS buffer to a final volume of 50 µL/test. Antibody panels are illustrated in table 1. Antibody cocktails were added, and cells were incubated for 20 min at 4°C. 2 mL FACS buffer was added to each tube to stop the reaction, followed by centrifugation at 350 g for 5 min, after which cells were resuspended in 150 µL fixation buffer (Biolegend) and 150 µL FACS buffer. Samples were stored at 4°C in the dark until flow cytometry collection.

**Table 1:** Flow cytometry staining panels

Panel	Antibody	Fluorophore	Source
Tissue panel 1	Epcam	BV650	Biolegend
	CD45	APCCy7	BD
	CD31	PE	Biolegend
	CD90	Vioblue	Miltenyi
	CD73	PercpCy5.5	Biolegend
	CD39	BV605	Biolegend
Tissue panel 2	CD45	APCCy7	BD
	CD3	AF700	Biolegend
	CD4	BV711	Biolegend
	CD8	FITC	Biolegend
	CD19	PE Dazzle	Biolegend
	CD56	PacBlue	Biolegend
	CD206	PE	Biolegend
	CD73	PercpCy5.5	Biolegend
	CD39	BV605	Biolegend
	CD103	APC	Biolegend
PBMC panel	CD45	APCCy7	BD
	CD3	AF700	Biolegend
	CD4	BV711	Biolegend
	CD8	FITC	Biolegend
	CD19	PE Dazzle	Biolegend
	CD56	PacBlue	Biolegend
	CD73	PercpCy5.5	Biolegend
	CD39	BV605	Biolegend
	CD103	APC	Biolegend

PBMC, peripheral blood mononuclear cell.

FACS data analysis was performed using FlowJo software. Gating strategy of tissue digest analysis is shown in online supplemental figure S17.

### mxIF staining

A TMA was constructed from consecutive patients undergoing curative resection surgery for NSCLC in a regional thoracic center. An experienced pathologist annotated each resection block and 1 mm cores were taken and embedded into the TMA. All patients were followed up to assess for relapse and death. Within our cohort, adjuvant chemotherapy was given to 20 patients in the form of cisplatin and vinorelbine or cisplatin and etoposide, and no patient received adjuvant immunotherapy in line with the standard of care at the time. The reasons for no adjuvant chemotherapy in this cohort include: not indicated (64), patient declined (33) oncology decision to decline (39) death or progression prior to therapy (3) and unknown (3).

The 4 µm sections were cut and slides were deparaffinised in Xylene and rehydrated in a series of ethanol dilutions. The following steps were performed on the Leica Bond automated staining robot. After initial heat-induced antigen retrieval (HIER) of 30 min at 100°C, tissue slides were exposed to multiple staining cycles each including a 30 min incubation with a protein block (Akoya), 1-hour incubation with the respective primary antibody, 30 min incubation with the secondary antibody (Akoya), 10 min incubation with the respective OPAL (Akoya) followed by 20 min incubation with AR6 buffer (Akoya) at 85°C prior to the next staining cycles and finally stained with fluorescent DAPI (Akoya) for 10 min. In between each step, slides were washed with bond wash for 5 min. The staining panel is illustrated in table 2.

**Table 2:** Multiplex immunofluorescence staining panel

Primary antibody	Primary antibody concentration	Source	OPAL pairing	Staining position
CD4 (EP204)	1:114 (70 ng/mL)	Cell signaling Technology	520	1
CD8 (4B11)	1:200	Thermo Fisher	570	2
PanCK (AE1/AE3)	1:200	Abcam	690	6
CD39 (EPR20627)	1:250	Abcam	620	5
CD73	1:500	Cambridge Bioscience	540	4
CD103 (EPR4166(2)	1:100	Abcam	650	3

A series of controls was performed to ensure correct staining. First, in addition to one fully stained multiplex slide, singleplex control slides were created for each marker, that underwent all HIER and washing steps, and only one respective staining cycle as well as the OPAL of the following cycle and DAPI. Signal intensities and staining patterns were highly comparable between multiplex and singleplex controls of each marker, respectively (online supplemental figure S18A), and primary antibodies were sufficiently stripped away in between staining cycles (online supplemental figure S18B). We could confirm that all signal intensities were in an acceptable range of 10–30 using InForm software. Furthermore, spectral library slides were created by staining consecutive NSCLC tumor tissue FFPE slides with primary PanCK antibody and each OPAL in the absence of any other staining, respectively (online supplemental figure S19).

### mxIF imaging

The appropriate exposure time for image acquisition was set for each fluorophore by autoexposing on multiple (5–10) tissue areas per batch. Following fluorescence whole slide scans, regions of interest were selected for multispectral imaging (MSI) at ×20 magnification, as scanning at ×40 magnification is time-consuming and does not improve image analysis.^44^

### mxIF analysis

MSI images were unmixed using representative snapshots of spectral library slides imaged at the same magnification and autofluorescence was isolated in InForm software. Unmixed images were exported and analyzed in Qupath^45^ (online supplemental figure S20A). Cell detection was performed using StarDist based on a watershed deep-learning algorithm and fluorescent threshold of DAPI nuclear staining.^46^ Following this, phenotyping was performed in a non-hierarchical manner by creating a composite classifier of single channel classifiers for each stain based on a fluorescent threshold that was adjusted for images if necessary. Ultimately, a machine learning algorithm was trained on multiple images to detect tumor and stroma areas (online supplemental figure S20B). Exported measurements included area measurements of tissue region and counts of cells that are positive for any combination of markers within these. Quantitative analysis was performed in Rstudio. Frequency was defined as percentage of positive cells within a cell type. Distribution was defined as percentage of the total of positive cells found within a cell type. Survival analysis was adjusted for age, sex, smoking, tumor size, adjuvant chemotherapy, frequency of PanCK^+^ cells, frequency of CD4^+^ cells overall, frequency of CD73^+^ among PanCK^+^ cells, and density of CD8^+^ cells in the tumor nest via Cox multivariate regression analysis using the coxph() function in R.

### Nanostring nCounter

NCL and tumor tissue samples from six patients were minced and digested as described above. Cells were stained for flow cytometry as described above using the antibody panel shown in table 3.

**Table 3:** Flow cytometry sorting staining panel

Antibody	Fluorophore	Source
CD45	APCCy7	BD
CD3	AF700	Biolegend
CD4	PE	Biolegend
CD8	APC	Biolegend
CD11b	FITC	Biolegend

CD4^+^ T cells from four paired and CD8^+^ T cells from five paired NCL and tumor tissue samples and one additional tumor tissue sample were collected on the FACS Aria Fusion into RNAse free eppendorfs (Qiagen), transported on ice and cell pellets were resuspended and lysed in RLT buffer (Qiagen) prior to freezing at −80°C. Analysis of a defined set of 780 genes (CAR T cell panel) was performed on the NanoString nCounter Analysis System. Only samples that passed nSolver QC were used for downstream analysis. This excluded one sample (CD4 NCL patient 2) due to low counts. Data from two separate nCounter runs were normalized in nSolver (Nanostring) using a calibration sample and normalized to HK genes selected using GeNorm algorithm. Normalized gene counts were used for Advanced Analysis in nSolver. Heatmaps of z-score of normalized counts of DEG (log2FC>/<0.5, p<0.05) were created using the pheatmap() function in R.

### Single cell RNA Sequencing data analysis

Single cell RNA sequencing data of T cells from human NSCLC tissue samples was downloaded from SCope (https://aertslab.org/).^12^ CD4^+^, CD8^+^, CD39^+^ and CD103^+^ T cells were defined by gene expression counts of CD4 >0, CD8a >0, ENTPD1 >0 and ITGAE >0, respectively. Differential expression analysis was performed in R using the DESeq2 package.^47^ Volcano plots were created using the EnhancedVolcano() function. GO biological processes analyses of upregulated genes in CD39^+^ CD4^+^ T cells (log2Fold change>1, padj.<0.1) and CD39^+^CD103^+^CD8^+^ T cells (log2Fold change>0.5, padj.<0.1) were performed using the enrichGO() function of the R package clusterProfiler (OrgDb=org.Hs.eg.db, ont=BP). GSEA of DEGs was performed using the gseGO() function of the R package clusterProfiler using gene sets obtained from GO biological processes database.

### TCGA data analysis

TCGA-LUAD and TCGA-LUSC datasets were used to analyze ENTPD1, ITGAE and NT5E expression and correlation to outcome. Preprocessed gene expression data (fragments per kilobase per million fragments, upper quartile normalized) of primary solid tumors and corresponding normal solid tissue as well as corresponding patient clinical data were downloaded using the Genomic Data Commons (GDC) data transfer tool. PFS data were downloaded from Liu *et al*.^48^ High/low expression of each marker was determined by the surv_cutpoint() function of the R survival package. Survival analysis was adjusted for age, gender, smoking and stage via Cox multivariate regression analysis using the coxph() function in R.

### INSPIRE survival analysis

RNA-seq data and survival data were downloaded from the Investigator-initiated Phase II Study of Pembrolizumab Immunological Response Evaluation (INSPIRE) study (NCT02644369).^27^ Preprocessed, batch normalized and log2 transformed gene expression data (TPM) were used to make groups of low/high ENTPD1 and ITGAE based on median cut-offs of this cohort.

## Data Availability

Data are available in a public, open access repository. Data are available on reasonable request. Targeted RNA Seq data is deposited in https://doi.org/10.7488/ds/7447. Flow cytometry and imaging data are available on request.
