# Supplementary material for: Location of CD39+ T cell subpopulations within tumors predict differential outcomes in non-small cell lung cancer
Source: J Immunother Cancer. 2023 Aug 30;11(8):e006770. doi: 10.1136/jitc-2023-006770 (PMC10471883; doi:10.1136/jitc-2023-006770)
Supplement: Supplementary data [file jitc-2023-006770supp001.pdf]

Supplementary Materials for

Location of CD39$^+$ T cell sub-populations within tumours predict differential outcomes in non-small cell lung cancer.

Lilian Koppensteiner, Layla Mathieson, Samuel Pattle, David A. Dorward, Richard O'Connor, Ahsan R. Akram

Supplementary Figures 1-20

Supplementary Tables 1-4

Location of CD39^{+} T cell sub-populations within tumours predict differential outcomes in non-small cell lung cancer.

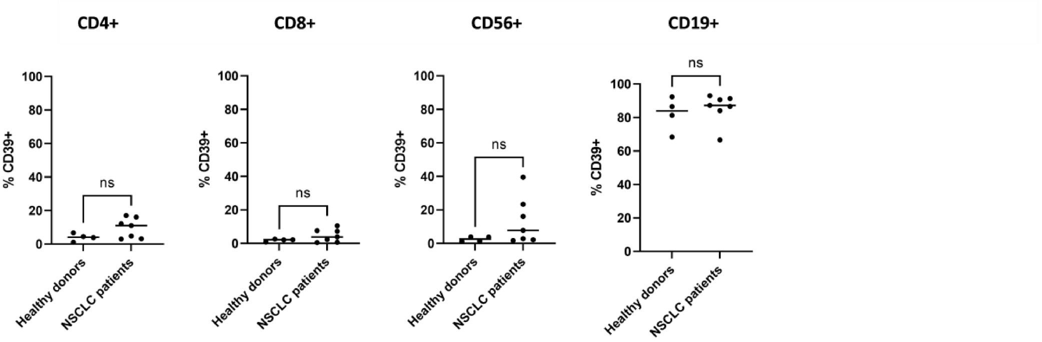

Figure S1 *Frequency of CD39^{+} amongst CD4^{+}, CD8^{+}, CD56^{+} and CD19^{+} cells from PBMCs of healthy donors and NSCLC patients. Unpaired T tests were used for comparing groups. (ns = not significant)*

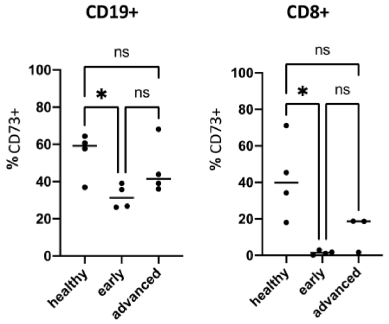

Figure S2 *Frequency of CD73^{+} amongst CD19^{+} and CD8^{+} cells from PBMCs of healthy donors and early and advanced NSCLC patients. One way ANOVAs with Tukeys multiple comparisons were used for comparing groups. (* p=<0.05, ** p=<0.01, ***p=<0.001)*

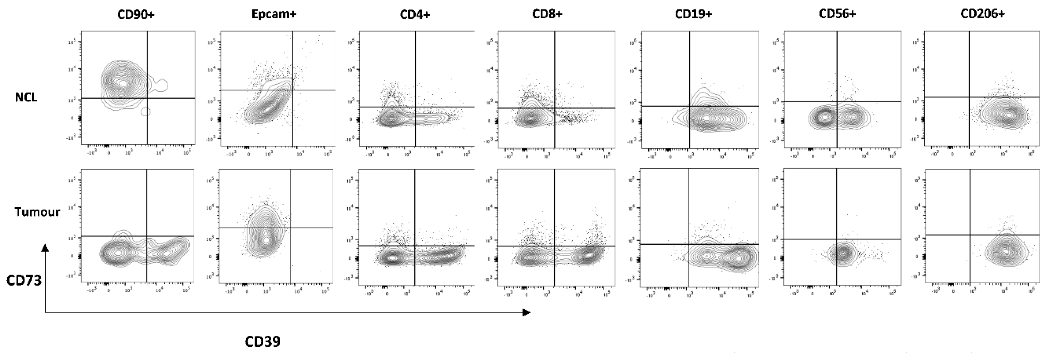

Figure S3 *Representative CD39 and CD73 flow cytometry staining of CD90^{+}, Epcam^{+}, CD4^{+}, CD8^{+}, CD19^{+}, CD56^{+} and CD206^{+} cells from NCL and tumour tissue.*

Location of CD39^{+} T cell sub-populations within tumours predict differential outcomes in non-small cell lung cancer.

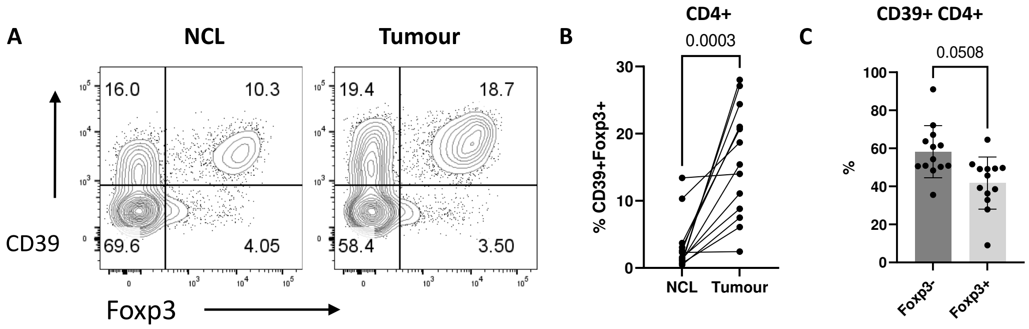

Figure S4 *A) Representative CD39 and FOXP3 flow cytometry staining of CD4^{+} T cells from a paired NCL and tumour tissue sample. B) Frequency of CD39^{+}Foxp3^{+} cells amongst CD4^{+} T cells in 13 paired NCL and tumour tissue samples. C) Frequency of FOXP3+ cells amongst CD39+ CD4+ T cells in 13 tumour tissue samples. Paired t tests were used for statistical analysis.*

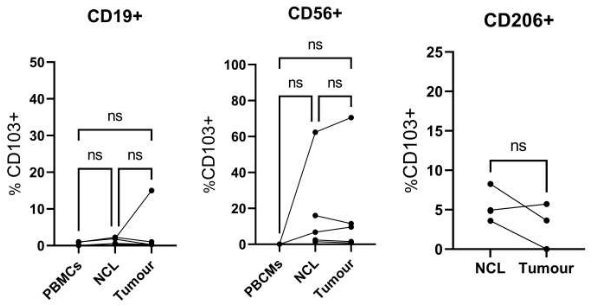

Figure S5 *Expression levels of CD103 shown as % positive within subpopulations of CD19^{+} and CD56^{+} cells from peripheral blood, NCL and tumour tissue and CD206^{+} cells from NCL and tumour tissue of early NSCLC patients. Paired t tests were used for statistical analysis of 2 groups, one way ANOVAs with Tukeys multiple comparisons were used for comparing 3 groups. (* p=<0.05, ** p=<0.01, ***p=<0.001)*

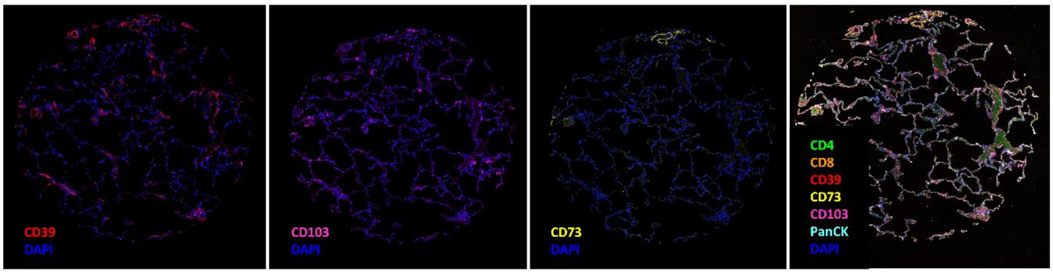

Figure S6 *Representative staining patterns of CD39, CD103 and CD73 in a NCL tissue sample.*

Location of CD39^{+} T cell sub-populations within tumours predict differential outcomes in non-small cell lung cancer.

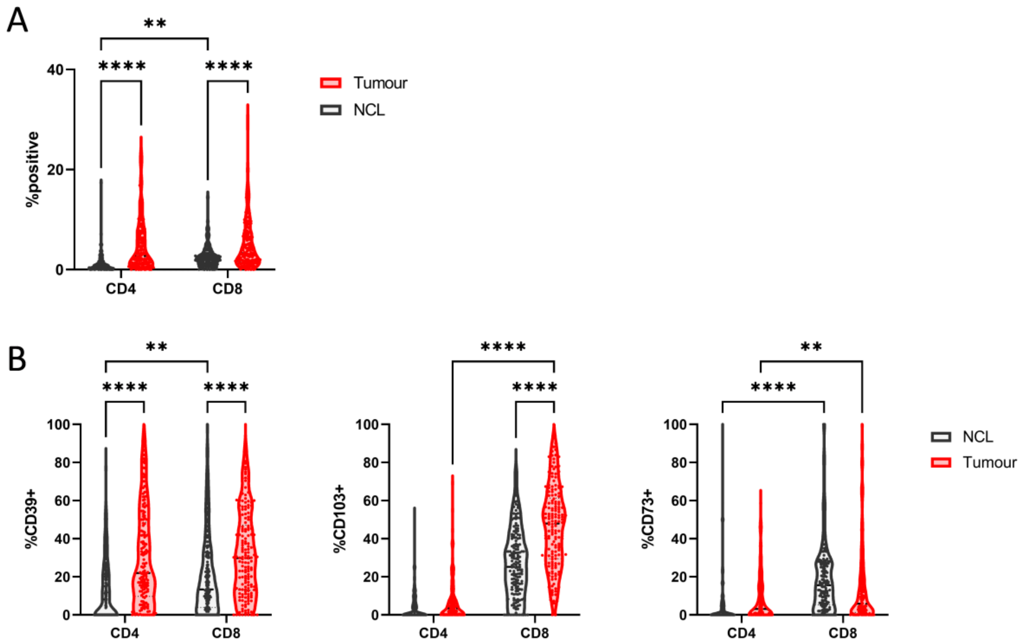

Figure S7 *(A) Frequency of CD4^{+} and CD8^{+} T cells in tumour compared to NCL tissue. N= 162 (B) Frequency of CD39^{+}, CD103^{+} and CD73^{+} cells within CD4^{+} and CD8^{+} T cells in NCL and tumour tissue from MxIF analysis of early untreated NSCLC patients N= 162 . Two-way ANOVAs were used to compare groups. (* p=<0.05, ** p=<0.01, ***p=<0.001)*

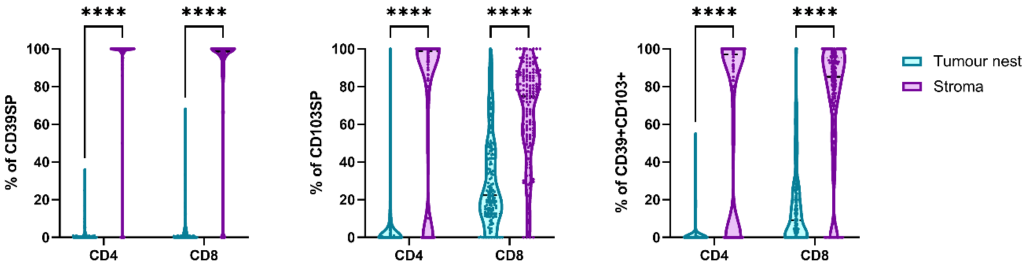

Figure S8 *Distribution of CD39SP, CD103SP and CD39^{+}CD103^{+} CD4^{+} and CD8^{+} T cells across tumour nest and stroma, respectively N= 162. Two-way ANOVAs were used to compare groups. (* p=<0.05, ** p=<0.01, ***p=<0.001)*

Location of CD39[+] T cell sub-populations within tumours predict differential outcomes in non-small cell lung cancer.

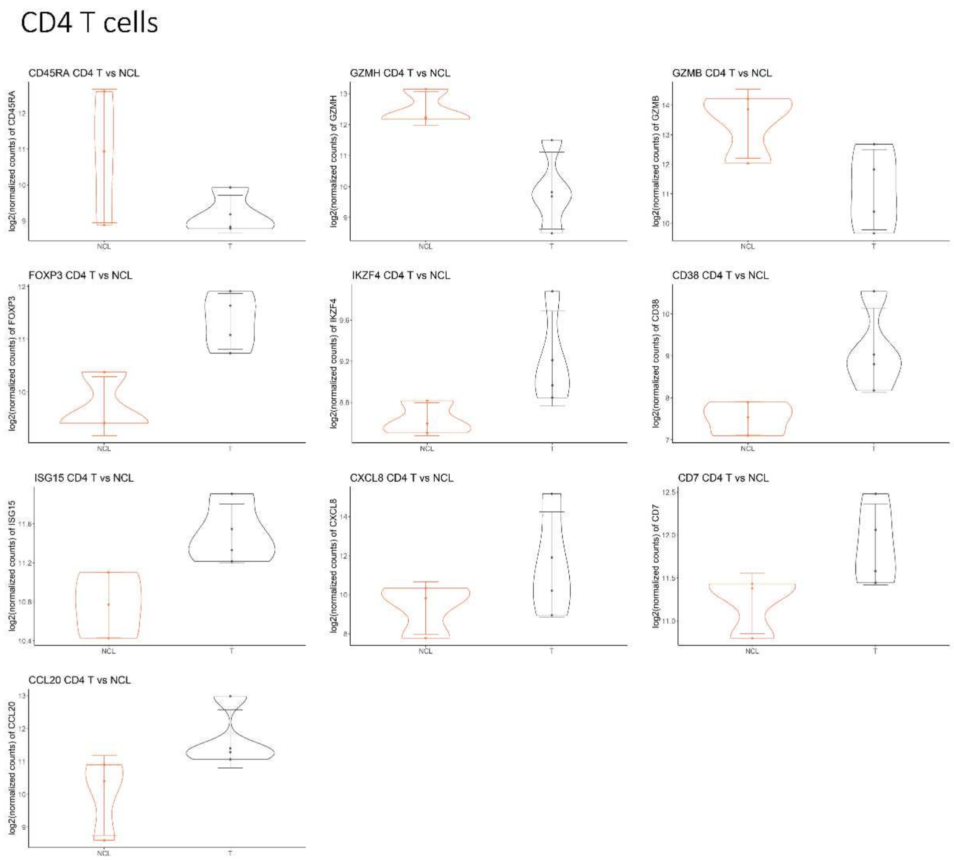

Figure S9 *Log2 of normalised counts of differentially expressed genes between CD4+ T cells from NCL and Tumour tissue from targeted RNA Seq analysis (Nanostring).*

Location of CD39[+] T cell sub-populations within tumours predict differential outcomes in non-small cell lung cancer.

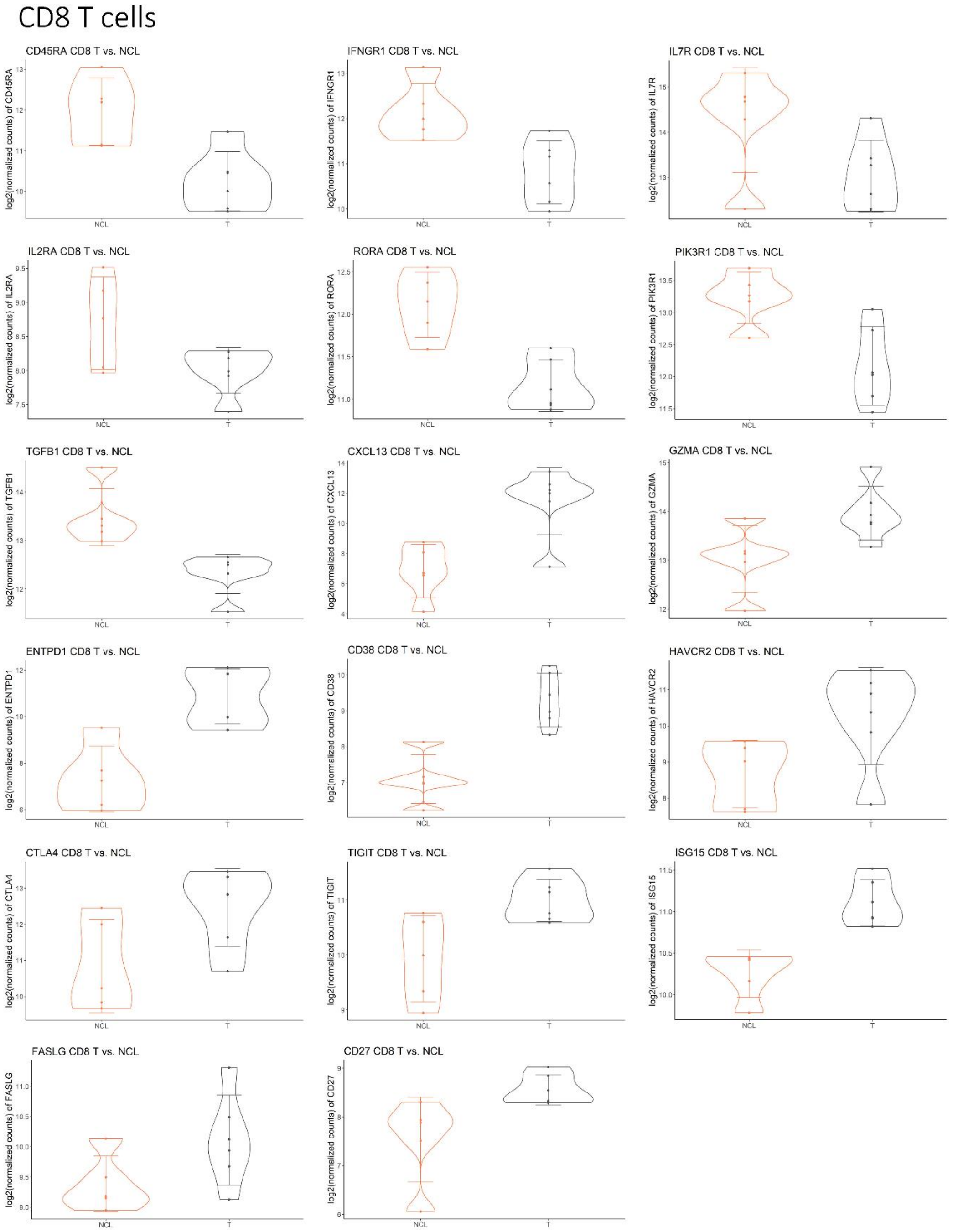

Figure S10 *Log2 of normalised counts of differentially expressed genes between CD8+ T cells from NCL and Tumour tissue from targeted RNA Seq analysis (Nanostring).*

Location of CD39[+] T cell sub-populations within tumours predict differential outcomes in non-small cell lung cancer.

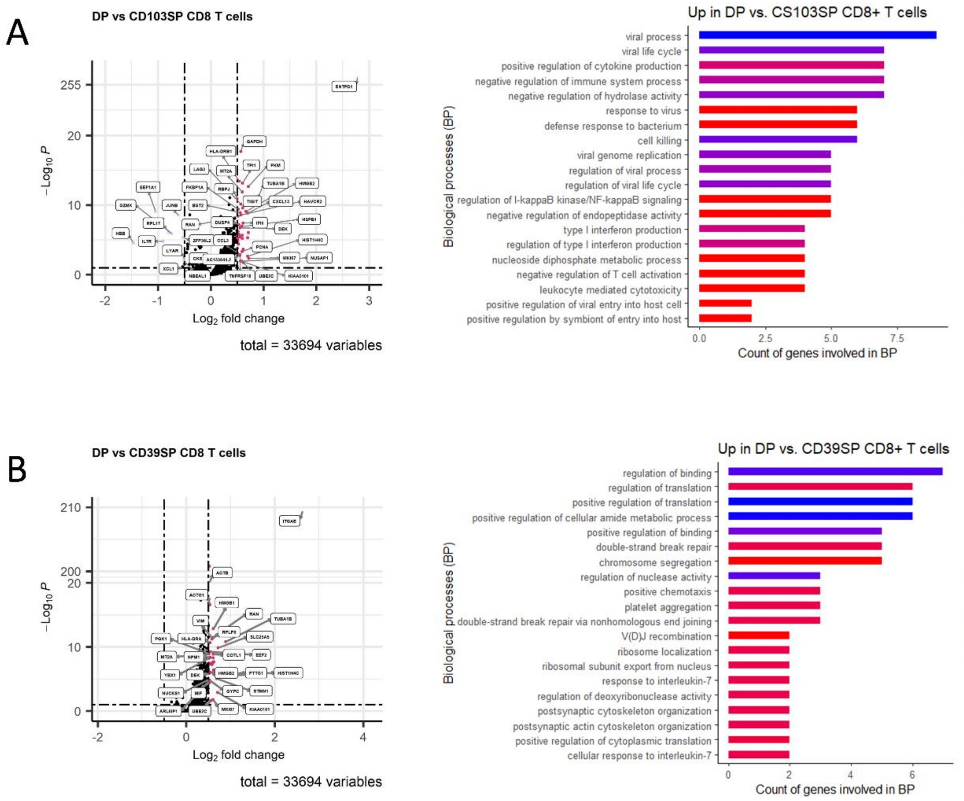

Figure S11 *(A) (left) Volcano plot showing DEG between CD39[+]CD103[+] and CD103SP CD8[+] T cells. (right) Bar plots of enrichment p values and gene count of respective gene sets of top 20 GO biological processes upregulated in CD39[+]CD103[+] compared to CD103SP CD8[+] T cells. (B) (left) Volcano plot showing DEG between CD39[+]CD103[+] and CD39SP CD8[+] T cells. (right) Bar plots of enrichment p values and gene count of respective gene sets of top 20 GO biological processes upregulated in CD39[+]CD103[+] compared to CD39SP CD8[+] T cells.*

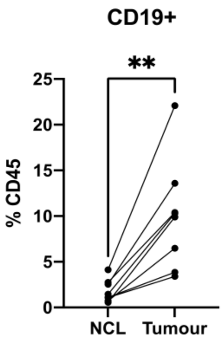

Figure S12 *Frequency of CD19[+] of CD45[+] cells from NCL and tumour tissue of early NSCLC patients. Paired t tests were used for statistical analysis. (* p=<0.05, ** p=<0.01, ***p=<0.001)*

Location of CD39^{+} T cell sub-populations within tumours predict differential outcomes in non-small cell lung cancer.

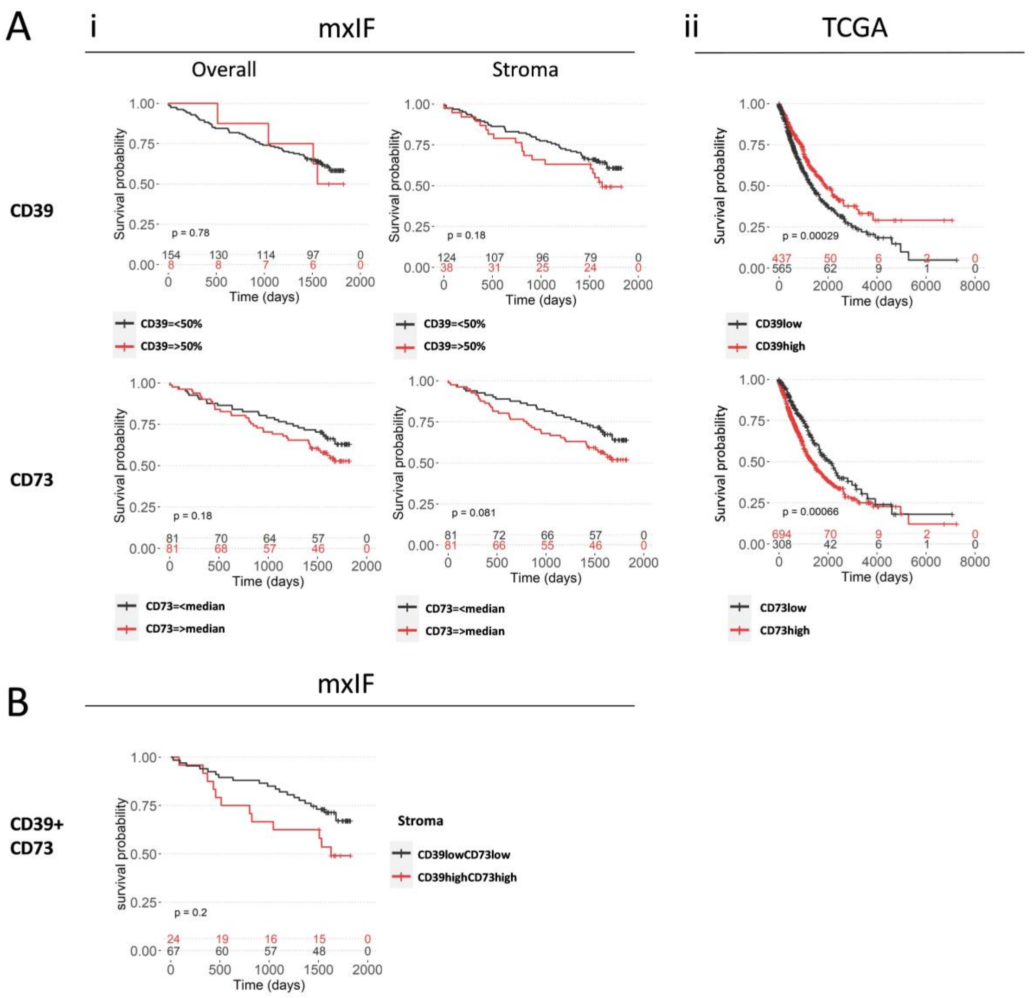

Figure S13 *(A) Kaplan Meier curves showing (i) 5 year OS based on stromal and intratumoural expression of CD39 (top panel) and CD73 (bottom panel) of mxIF data of early untreated NSCLC patients (n=162) and (ii) 19 year OS probability based on high/low CD39 (top) and CD73 (bottom) of TCGA data. (B) 5 year OS based on combined stromal CD39 and CD73 expression of mxIF data. Log Rank tests were used for survival analysis*

Koppensteiner L, *et al. J Immunother Cancer* 2024; 11:e006770. doi: 10.1136/jitc-2023-006770

Location of CD39$^+$ T cell sub-populations within tumours predict differential outcomes in non-small cell lung cancer.

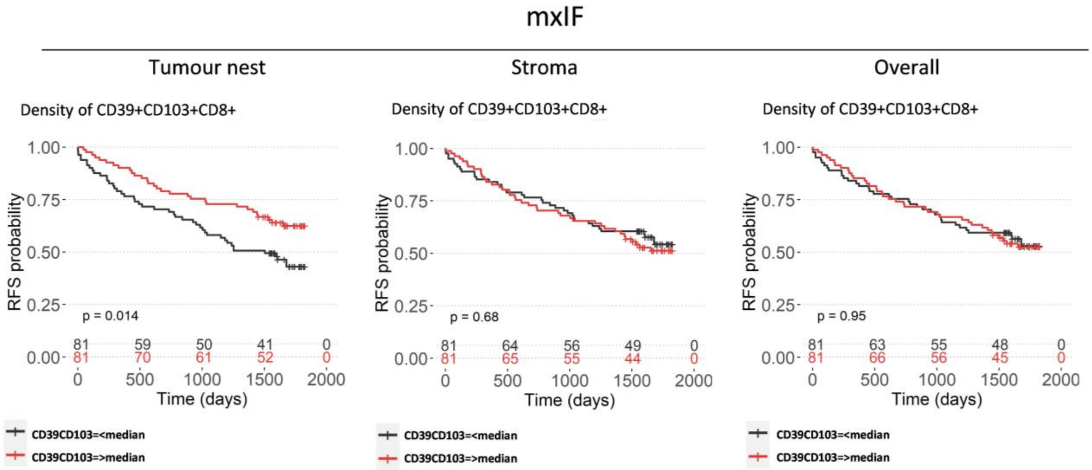

Figure S14 *Effect of spatial expression patterns of CD39*$^+$*CD103*$^+$*CD8*$^+$ *T cells on RFS probability at 5 years in early untreated NSCLC patients (n=162). Log Rank tests were used for survival analysis*

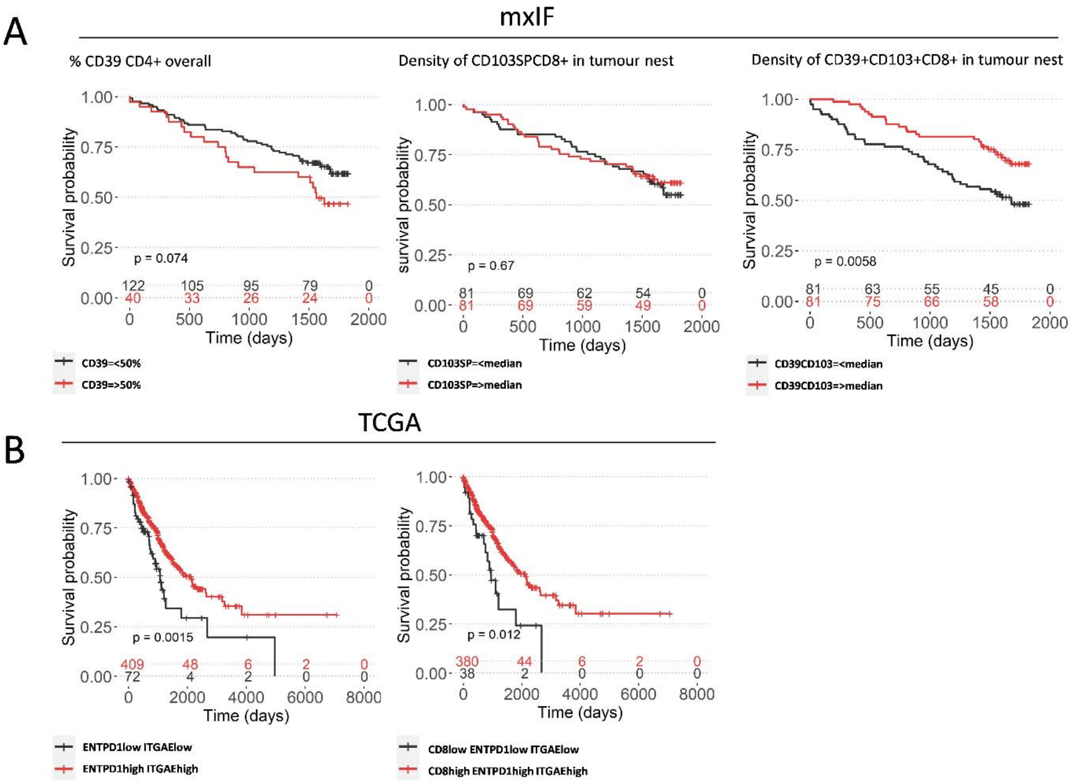

Figure S15 *Kaplan Meier curves showing (A) 5 year OS based on frequency of CD39*$^+$ *within CD4*$^+$ *T cells overall (left) and density of CD103SP CD8*$^+$ *T cells (centre) and CD39*$^+$*CD103*$^+$ *CD8*$^+$ *T cells in the tumour nest (right). (B) 19 year OS probability based on high/low ENTPD1 and ITGAE (left) and CD8, ENTPD1 and ITGAE (right) of TCGA data. Log Rank tests were used for survival analysis*

Koppensteiner L, *et al. J Immunother Cancer* 2024; 11:e006770. doi: 10.1136/jitc-2023-006770

Location of CD39$^+$ T cell sub-populations within tumours predict differential outcomes in non-small cell lung cancer.

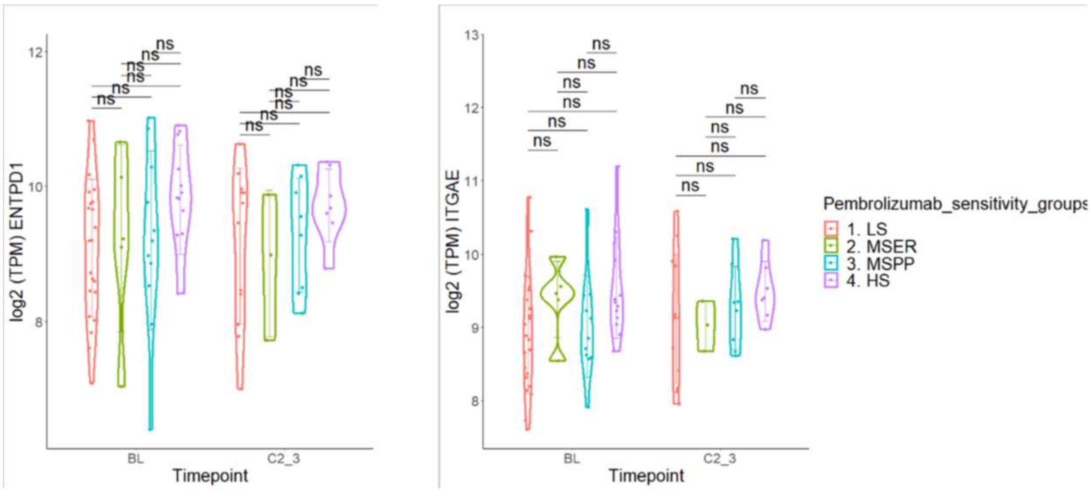

Figure S16 *Comparison of ENTPD1 (left) and ITGAE (right) expression levels between baseline prior to therapy (BL) and following 2-3 cycles of pembrolizumab (C2_3) and pembrolizumab sensitivity groups as defined by Yang et al.* [25]

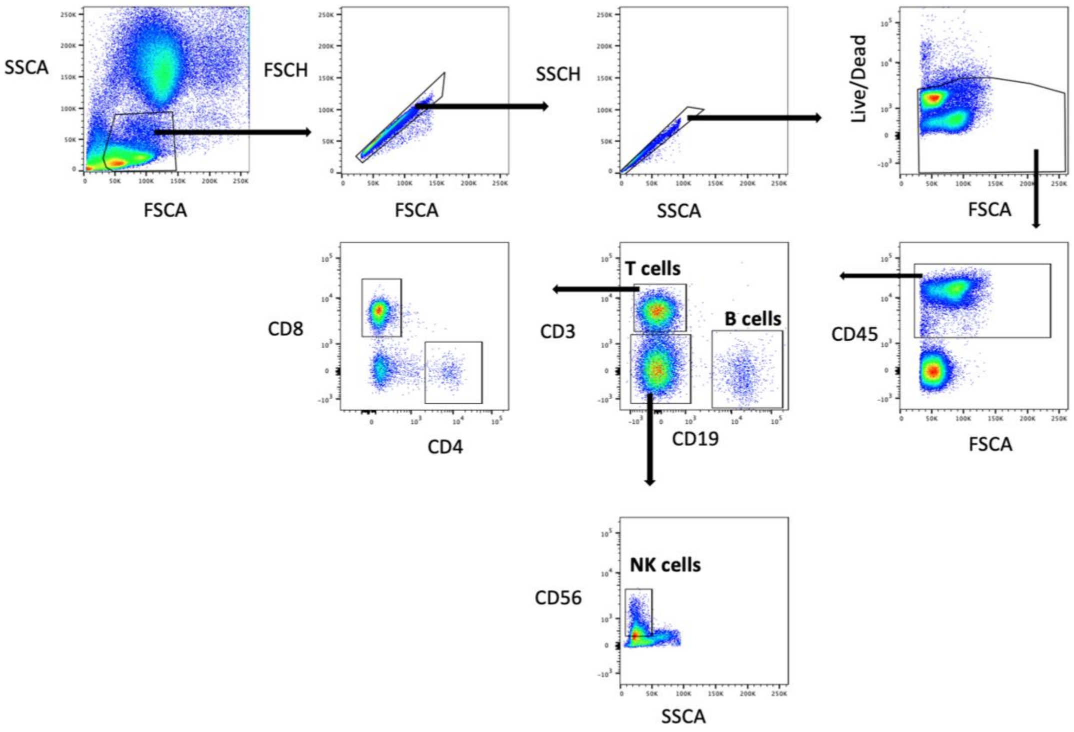

Figure S17 Gating strategy for tissue digest. *We first set a cell-gate to remove debris, then gate on single cells, and live cells. Within live single cells, we gate on EPCAM$^+$ and CD45$^+$ cells. Within the Epcam$^-$CD45$^-$ population, we gate on CD90$^+$ cells. Within CD45$^+$ cells, we gate on CD19$^+$cells and CD3$^+$cells. Within the CD19$^-$CD3$^-$ cells, we gate for NK cells and macrophages by selecting CD56$^+$ and CD206$^+$, respectively. Within CD3$^+$T cells we distinguish between CD4$^+$and CD8$^+$T cells.*

Koppensteiner L, et al. J Immunother Cancer 2024; 11:e006770. doi: 10.1136/jitc-2023-006770

Location of CD39[+] T cell sub-populations within tumours predict differential outcomes in non-small cell lung cancer.

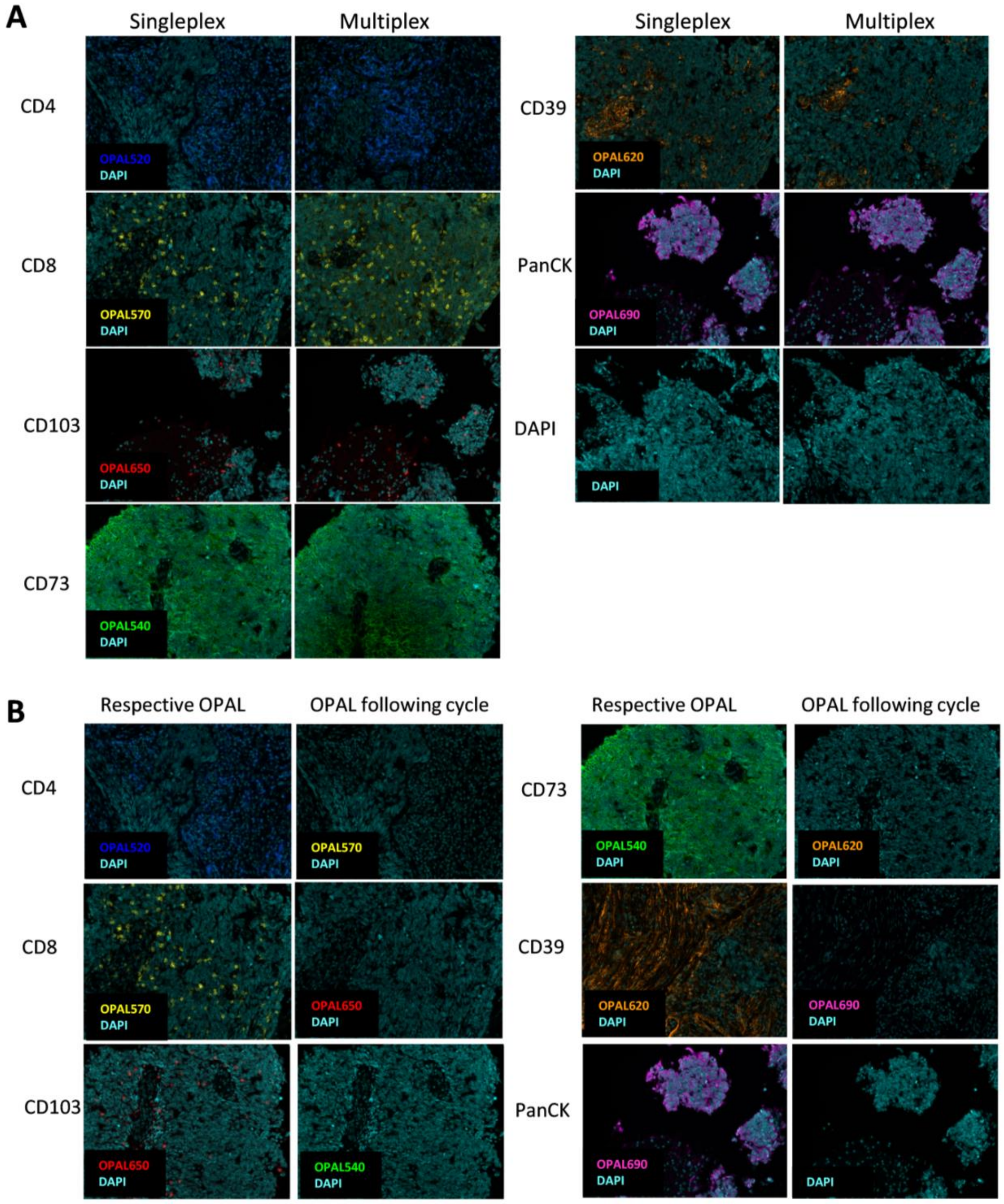

Figure S18 *A) Singleplex controls: Images show fluorescent single stain controls (left) and the respective channel in the corresponding tissue region of a slide stained with the full multiplex panel (right) to compare staining intensity and pattern. B) Drop out controls were created to confirm that primary antibodies are completely stripped away by heating steps in between staining cycles to prevent signal crosstalk. Images show OPAL signal of the full staining cycle (left) and the OPAL signal of the following staining cycle (right), for each staining cycle respectively.*

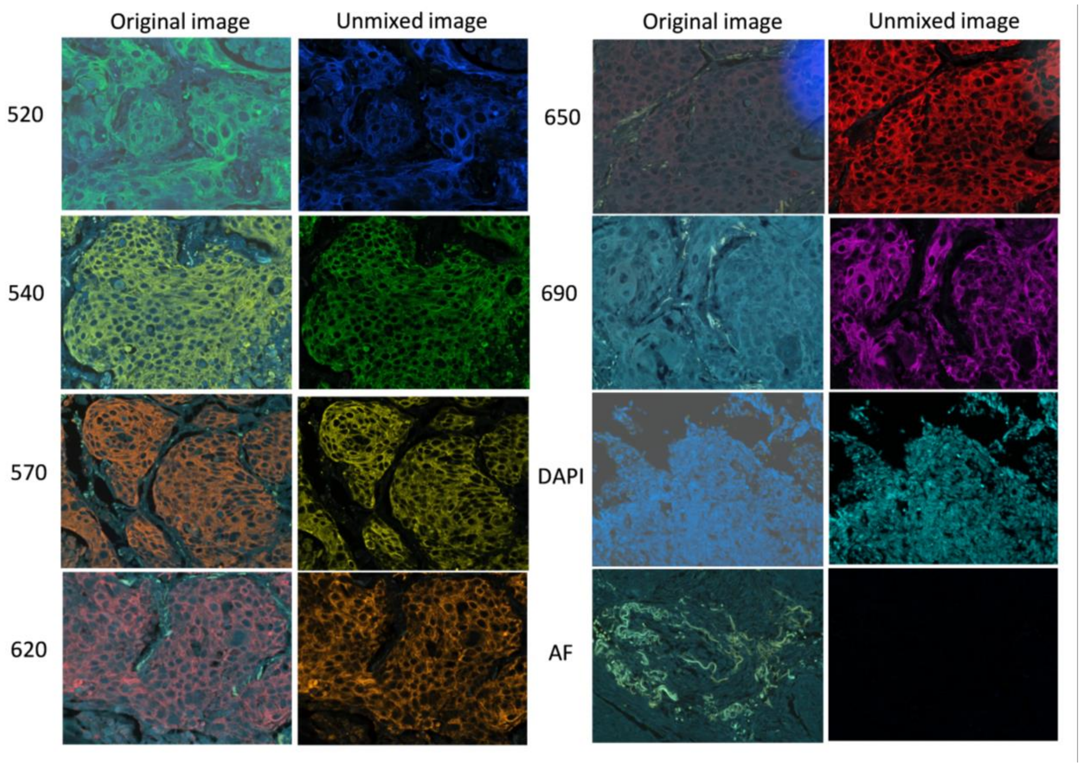

Figure S19 *Spectral library slides used for spectral unmixing, created by staining consecutive NSCLC tumour tissue slides with primary PanCK antibody and each OPAL (at 1:200 dilutions), in the absence of any other staining.*

Location of CD39$^+$ T cell sub-populations within tumours predict differential outcomes in non-small cell lung cancer.

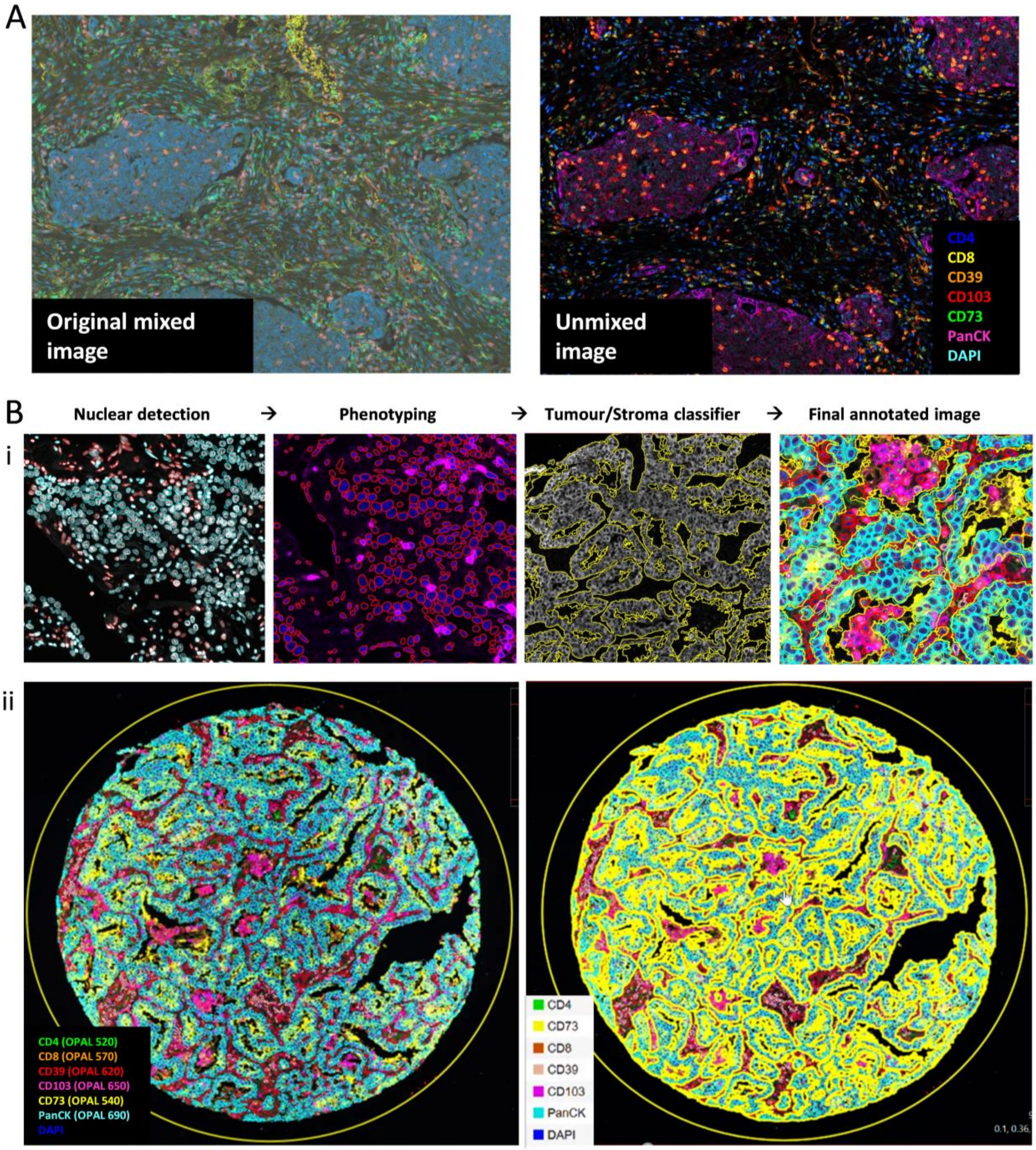

Figure S20 MxIF image analysis workflow. *(A) Example image of NSCLC tumour tissue stained with full multiplex panel showing original mixed image (left) and spectrally unmixed image (right). (B) (i) Overview of optimised image analysis workflow in Qupath and (ii) example of original unmixed staining (left) and image showing tumour stroma classified and phenotype annotated image.*

Location of CD39^{+} T cell sub-populations within tumours predict differential outcomes in non-small cell lung cancer.

A) Tumour TMA patients

Variable		N=162 n(%)
**Sex**	f	93 (57.4)
	m	69 (42.6)
**Histology**	Adenocarcinoma	89 (54.9)
	Large cell	6 (3.7)
	Mixed	6 (3.7)
	Neuroendocrine	4 (2.5)
	Squamous	57 (35.2)
**Age**	Mean (SD)	68.3 (10.2)
**Adjuvant chemotherapy**	no	142 (87.7)
	yes	20 (12.3)

B) NCL TMA patients

Variable		N=163 n(%)
**Sex**	f	93 (57.1)
	m	70 (42.9)
**Age**	Mean (SD)	68.5 (10.2)

Table S1 *Patient demographics of tumour TMA (A) and NCL TMA (B) samples.*

Location of CD39^{+} T cell sub-populations within tumours predict differential outcomes in non-small cell lung cancer.

A mxIF

RFS

	Univariate				Multivariate			
variable	HR	95% lower CI	95% upper CI	p-value	HR	95% lower CI	95% upper CI	p-value
adjuvant chemotherapy (yes)	0.8276654	0.3975663	1.7230582	0.61314673	0.6779621	0.3122601	1.471954	0.3258033
age	1.0224902	0.9951323	1.0506002	0.10798367	1.0219947	0.9944638	1.050288	0.1184037
sex (male)	1.3511124	0.8584424	2.1265315	0.19346846	1.4918185	0.8968626	2.481453	0.1233924
smoking (packyears)	0.9972449	0.9885939	1.0059716	0.53485149	0.9944841	0.9856678	1.003379	0.2234463
smoking (yes)	0.8287818	0.5232172	1.3127996	0.42357254	0.8275465	0.4952496	1.382804	0.4699062
Stroma CD39(low)	0.6031998	0.3666519	0.9923582	0.04657277	0.5954574	0.3467294	1.022612	0.0602547
tumour size	1.0306978	0.8954406	1.1863857	0.67356183	1.0535732	0.8911494	1.245601	0.5412575

B

RFS

	Univariate				Multivariate			
variable	HR	95% lower CI	95% upper CI	p-value	HR	95% lower CI	95% upper CI	p-value
adjuvant chemotherapy (yes)	0.8276654	0.3975663	1.7230582	0.61314673	0.7788125	0.3622029	1.674611	0.52217325
age	1.0224902	0.9951323	1.0506002	0.10798367	1.0206450	0.9920054	1.050111	0.15936316
sex (male)	1.3511124	0.8584424	2.1265315	0.19346846	1.4331843	0.8659727	2.371919	0.16147308
smoking (packyears)	0.9972449	0.9885939	1.0059716	0.53485149	0.9937549	0.9850039	1.002584	0.16507922
smoking (yes)	0.8287818	0.5232172	1.3127996	0.42357254	0.8354498	0.5020311	1.390305	0.48902246
Stroma CD73(low)	0.5933579	0.3744312	0.9402891	0.02627859	0.6512673	0.3945141	1.075118	0.09358962
tumour size	1.0306978	0.8954406	1.1863857	0.67356183	1.0634071	0.9039438	1.251001	0.45829337

C

RFS

	Univariate				Multivariate			
variable	HR	95% lower CI	95% upper CI	p-value	HR	95% lower CI	95% upper CI	p-value
adjuvant chemotherapy (yes)	0.8276654	0.3975663	1.723058	0.61314673	0.7463904	0.3443408	1.617870	0.45865273
age	1.0224902	0.9951323	1.050600	0.10798367	1.0214027	0.9923893	1.051264	0.14976968
CD73(low)	0.6455078	0.4081936	1.020791	0.06121193	0.6253922	0.3805704	1.027708	0.06400903
PanCK (low)	1.0635999	0.6760937	1.673207	0.78967852	0.9378056	0.5707671	1.540872	0.79991895
sex (male)	1.3511124	0.8584424	2.126531	0.19346846	1.5094185	0.9134380	2.494252	0.10813019
smoking (packyears)	0.9972449	0.9885939	1.005972	0.53485149	0.9932786	0.9843904	1.002247	0.14141376
smoking (yes)	0.8287818	0.5232172	1.312800	0.42357254	0.8214942	0.4922458	1.370967	0.45175274
tumour size	1.0306978	0.8954406	1.186386	0.67356183	1.0686581	0.9087430	1.256714	0.42202714

Table S2 *Univariate and multivariate cox regression analysis of A) adjuvant chemotherapy, age, sex, smoking, tumour size, and stromal CD39 (</>50% expression), B) adjuvant therapy, age, sex, smoking, tumour size, and stromal CD73 (</> median % expression), and C) adjuvant chemotherapy, age, sex, smoking, tumour size, overall PanCK (>/< % expression) and overall CD73 (</> median % expression).*

Koppensteiner L, *et al. J Immunother Cancer* 2024; 11:e006770. doi: 10.1136/jitc-2023-006770

Location of CD39^{+} T cell sub-populations within tumours predict differential outcomes in non-small cell lung cancer.

A TCGA

PFS

	Univariate				Multivariate			
**variable**	**HR**	**95% lower CI**	**95% upper CI**	**p-value**	**HR**	**95% lower CI**	**95% upper CI**	**p-value**
age	1.008590	0.9990517	1.018220	0.0776846870	1.0139905	1.0027200	1.025388	0.01483920
ENTPD1(low)	1.286771	1.0769104	1.537528	0.0055090384	1.1992802	0.9737748	1.477008	0.08728340
gender (male)	1.090735	0.9116303	1.305029	0.3426145883	1.1459634	0.9228326	1.423045	0.21752991
smoking (pack years)	1.000383	0.9970711	1.003705	0.8210521888	0.9992541	0.9958550	1.002665	0.66775651
stage (IV)	2.003133	1.3261168	3.025782	0.0009626648	1.7071028	1.0288367	2.832520	0.03845226

B

PFS

	Univariate				Multivariate			
**variable**	**HR**	**95% lower CI**	**95% upper CI**	**p-value**	**HR**	**95% lower CI**	**95% upper CI**	**p-value**
age	1.0085903	0.9990517	1.0182199	0.077684687028	1.0140652	1.0029038	1.0253509	0.01338150813
gender (male)	1.0907353	0.9116303	1.3050286	0.342614588252	1.2966768	1.0414179	1.6145014	0.02018986494
NT5E(low)	0.6241213	0.5121013	0.7606452	0.000003003285	0.6212947	0.4962400	0.7778638	0.00003315158
smoking (pack years)	1.0003827	0.9970711	1.0037054	0.821052188819	0.9992055	0.9958071	1.0026155	0.64749005866
stage (IV)	2.0031327	1.3261168	3.0257823	0.000962664803	1.5927581	0.9591527	2.6449161	0.07205109349

Table S3 *Univariate and multivariate cox regression analysis of PFS based on age, gender, smoking, stage and ENTPD1 (low/ high) and NT5E (low/high).*

Location of CD39$^+$ T cell sub-populations within tumours predict differential outcomes in non-small cell lung cancer.

A mxIF

RFS

	Univariate				Multivariate			
**variable**	**HR**	**95% lower CI**	**95% upper CI**	**p-value**	**HR**	**95% lower CI**	**95% upper CI**	**p-value**
adjuvant chemotherapy (yes)	0.8276654	0.3975663	1.7230582	0.613146735	0.7108837	0.3318618	1.5227891	0.379958595
age	1.0224902	0.9951323	1.0506002	0.107983670	1.0227905	0.9951378	1.0512116	0.107086163
CD39% CD4+ (low)	0.4881926	0.3030565	0.7864274	0.003202613	0.4902862	0.2920647	0.8230388	0.007000307
CD4 (low)	0.9388715	0.5967884	1.4770389	0.784976464	1.0848884	0.6603162	1.7824534	0.747735169
sex (male)	1.3511124	0.8584424	2.1265315	0.193468463	1.4008577	0.8328899	2.3561367	0.203843616
smoking (packyears)	0.9972449	0.9885939	1.0059716	0.534851485	0.9930623	0.9838410	1.0023701	0.143573634
smoking (yes)	0.8287818	0.5232172	1.3127996	0.423572536	0.8600921	0.5084013	1.4550679	0.574225289
tumour size	1.0306978	0.8954406	1.1863857	0.673561831	1.0359158	0.8764221	1.2244345	0.679132844

B

RFS

	Univariate				Multivariate			
**variable**	**HR**	**95% lower CI**	**95% upper CI**	**p-value**	**HR**	**95% lower CI**	**95% upper CI**	**p-value**
adjuvant chemotherapy (yes)	0.8276654	0.3975663	1.723058	0.61314673	0.8797140	0.4000731	1.934388	0.749890217
age	1.0224902	0.9951323	1.050600	0.10798367	1.0243047	0.9943543	1.055157	0.112732573
frequency of CD73+ amongst PanCK+ cells (low)	0.7820100	0.4967229	1.231149	0.28827773	0.7374177	0.4304423	1.263316	0.267441043
sex (male)	1.3511124	0.8584424	2.126531	0.19346846	1.4349872	0.8727161	2.359517	0.154623402
smoking (packyears)	0.9972449	0.9885939	1.005972	0.53485149	0.9945152	0.9855660	1.003546	0.233052931
smoking (yes)	0.8287818	0.5232172	1.312800	0.42357254	0.8222360	0.4913195	1.376034	0.456278277
tumour nest CD39+CD103+CD8 density(low)	1.7806789	1.1213819	2.827598	0.01446436	2.3786143	1.3168569	4.296447	0.004074121
tumour nest CD8 density (low)	0.9472740	0.6023227	1.489780	0.81462314	0.7265614	0.3885379	1.358661	0.317199462
tumour size	1.0306978	0.8954406	1.186386	0.67356183	1.0427755	0.8927688	1.217987	0.597100295

Table S4 *Univariate and multivariate cox regression analysis of A) adjuvant chemotherapy, age, sex, smoking, tumour size, CD4$^+$ T cells (</> median % expression) and CD39$^+$CD4$^+$ (</> median % expression) and B) adjuvant chemotherapy, age, sex, smoking, tumour size, CD8$^+$ T cells (density in tumour nest </> median), frequency of CD73+ amongst PanCK+ cells (</> median) and CD39$^+$CD103$^+$CD8$^+$ T cells (density in tumour nest </> median)*

Koppensteiner L, et al. J Immunother Cancer 2024; 11:e006770. doi: 10.1136/jitc-2023-006770
